# Supplementary material for: Secondary analysis of a James Lind Alliance priority setting partnership to facilitate knowledge translation in degenerative cervical myelopathy (DCM): insights from AO Spine RECODE-DCM
Source: BMJ Open. 2023 Jul 18;13(7):e064296. doi: 10.1136/bmjopen-2022-064296 (PMC10357680; doi:10.1136/bmjopen-2022-064296)

Supplementary Materials

Supplementary Material 1: Proportion of respondents of male sex submitting answered (Grey) and unanswered research questions (Orange), by principal stakeholder group and overall (black and red). Whilst male PwCM or their supporters were less likely to submit an answered research question, overall this association changed. It is likely this was driven by an interaction with Spinal Surgeons, who were almost universally male.

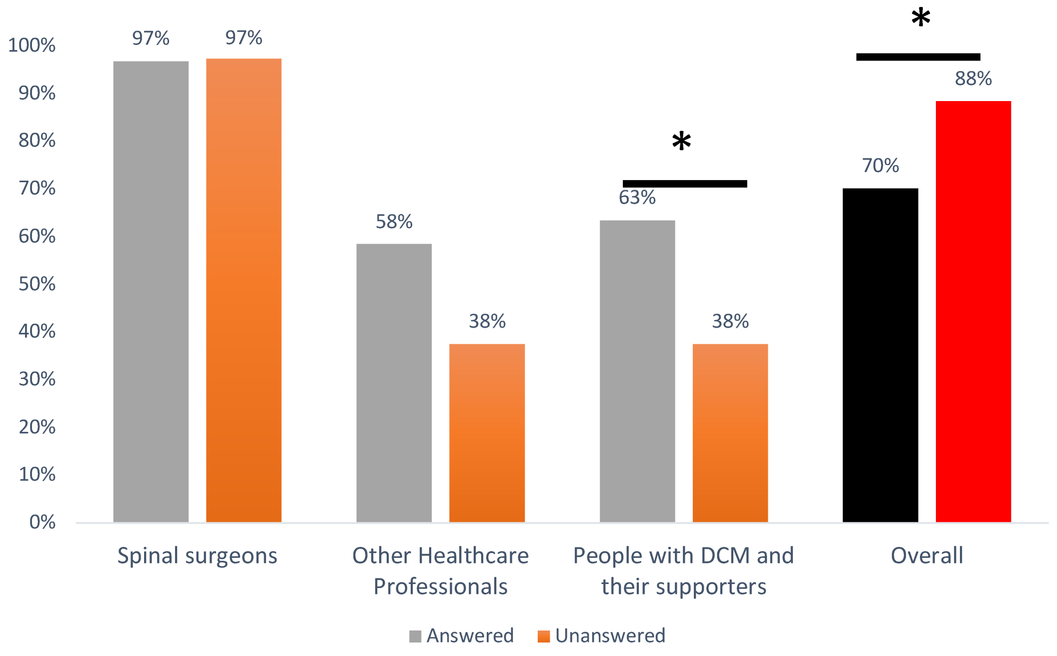

Supplement: Supplementary data [file bmjopen-2022-064296supp001.pdf]
