# Supplementary material for: Secondary analysis of a James Lind Alliance priority setting partnership to facilitate knowledge translation in degenerative cervical myelopathy (DCM): insights from AO Spine RECODE-DCM
Source: BMJ Open. 2023 Jul 18;13(7):e064296. doi: 10.1136/bmjopen-2022-064296 (PMC10357680; doi:10.1136/bmjopen-2022-064296)
Supplement: Author's manuscript [file bmjopen-2022-064296.draft_revisions.pdf]

BMJ Open is committed to open peer review. As part of this commitment we make the peer review history of every article we publish publicly available.

When an article is published we post the peer reviewers' comments and the authors' responses online. We also post the versions of the paper that were used during peer review. These are the versions that the peer review comments apply to.

The versions of the paper that follow are the versions that were submitted during the peer review process. They are not the versions of record or the final published versions. They should not be cited or distributed as the published version of this manuscript.

BMJ Open is an open access journal and the full, final, typeset and author-corrected version of record of the manuscript is available on our site with no access controls, subscription charges or pay-per-view fees (http://bmjopen.bmj.com).

If you have any questions on BMJ Open's open peer review process please email info.bmjopen@bmj.com

BMJ Open

Leveraging a James Lind Alliance priority setting partnership to facilitate knowledge translation in degenerative cervical myelopathy (DCM): Insights from AO Spine RECODE-DCM

Journal:	*BMJ Open*
Manuscript ID	bmjopen-2022-064296
Article Type:	Original research
Date Submitted by the Author:	14-Aug-2022
Complete List of Authors:	Davies, Benjamin; University of Cambridge Neurosurgery, Department of Clinical Neurosurgery; University of Cambridge, University of Cambridge<br>Brannigan , Jamie ; University of Cambridge School of Clinical Medicine,<br>Mowforth, Oliver; University of Cambridge, Department of Academic Neurosurgery<br>Khan, Danyal; University College London,<br>McNair, Angus; University of Bristol, Centre for Surgical Research, Bristol Medical School: Population Health Sciences; North Bristol NHS Trust, GI Surgery<br>Tetreault, Lindsay; University of Toronto, Department of Surgery; University College Cork, Department of Medicine<br>Sadler, Iwan; Myelopathy.org<br>Sarewitz, Ellen; Myelopathy.org<br>Aarabi, Bizhan; University of Maryland Baltimore, Division of Neurosurgery,<br>Kwon, Brian; University of British Columbia, Division of Spine Surgery, Vancouver General Hospital<br>Gronlund, Toto ; National Institute for Health Research, University of Southampton, Southampton, UK<br>Rahimi-Movaghar, Vafa; Sina Trauma and Surgery Research Center, Academic Department of Neurological Surgery<br>Zipser, Carl Moritz; University Hospital Balgrist, Neurology<br>Hutchinson, Peter; University of Cambridge, Academic Neurosurgery<br>Kurpad, Shekar; Medical College of Wisconsin, Division of Neurosurgery<br>Harrop, James; Jefferson Health System,<br>Wilson, Jefferson R.; University of Toronto<br>Guest, James; University of Miami Miller School of Medicine, Department of Neurological Surgery<br>Fehlings, Michael G.; Toronto Western Hospital,<br>Kotter, Mark R.N.; University of Cambridge, Department of Clinical Neurosurgery; University of Cambridge
Keywords:	NEUROSURGERY, Spine < ORTHOPAEDIC & TRAUMA SURGERY, SURGERY

I, the Submitting Author has the right to grant and does grant on behalf of all authors of the Work (as defined in the below author licence), an exclusive licence and/or a non-exclusive licence for contributions from authors who are: i) UK Crown employees; ii) where BMJ has agreed a CC-BY licence shall apply, and/or iii) in accordance with the terms applicable for US Federal Government officers or employees acting as part of their official duties; on a worldwide, perpetual, irrevocable, royalty-free basis to BMJ Publishing Group Ltd ("BMJ") its licensees and where the relevant Journal is co-owned by BMJ to the co-owners of the Journal, to publish the Work in this journal and any other BMJ products and to exploit all rights, as set out in our licence.

The Submitting Author accepts and understands that any supply made under these terms is made by BMJ to the Submitting Author unless you are acting as an employee on behalf of your employer or a postgraduate student of an affiliated institution which is paying any applicable article publishing charge ("APC") for Open Access articles. Where the Submitting Author wishes to make the Work available on an Open Access basis (and intends to pay the relevant APC), the terms of reuse of such Open Access shall be governed by a Creative Commons licence – details of these licences and which Creative Commons licence will apply to this Work are set out in our licence referred to above.

Other than as permitted in any relevant BMJ Author's Self Archiving Policies, I confirm this Work has not been accepted for publication elsewhere, is not being considered for publication elsewhere and does not duplicate material already published. I confirm all authors consent to publication of this Work and authorise the granting of this licence.

For peer review only

Leveraging a James Lind Alliance priority setting partnership to facilitate knowledge translation in degenerative cervical myelopathy (DCM): Insights from AO Spine RECODE-DCM

Benjamin M. Davies M.R.C.S, B.Sc. [1], Jamie F.M. Brannigan B.A.(Hons).[1], Oliver D. Mowforth B.A.(Hons). M.B. BChir. [1], Danyal Z. Khan MB, BCh, BAO [1], Angus G.K. McNair, F.R.C.S Ph.D FHEA[2], Lindsay Tetreault Ph.D.[3], Iwan Sadler [4], Ellen Sarewitz M.A [5], Bizhan Aarabi MD [6], Brian K Kwon M.D. Ph.D F.R.C.S.C [7], Toto Gronlund BSc, MSc [8], Vafa Rahimi-Movaghar MD[9], Carl Moritz Zipser MD FEBN[10], Peter J.A. Hutchinson Ph.D F.R.C.S FMedSci[1], Shekar N Kurpad M.D Ph.D[11], James Harrop M.D. [12], Jefferson R Wilson M.D. Ph.D F.R.C.S.C [3], James D. Guest, MD PhD FACS [13], Michael G. Fehlings M.D. Ph.D F.R.C.S.C [3], Mark R.N. Kotter M.D. M.Phil. Ph.D. [1]

1) Academic Neurosurgery Unit, Department of Clinical Neurosurgery, University of Cambridge, Cambridge, UK
2) Center for Surgical Research, Bristol Medical School: Population Health Sciences, University of Bristol, UK
3) Division of Neurosurgery and Spine Program, Department of Surgery, University of Toronto, Toronto, Ontario, Canada
4) Myelopathy.org, Cambridge, United Kingdom. (Registered Charity England and Wales, No 1178673)
5) Person with DCM and Director of Goffin Consultancy, Healthcare Consultants.
6) Division of Neurosurgery, Shock Trauma, University of Maryland, Baltimore, Maryland, USA
7) Division of Spine Surgery, Vancouver General Hospital, University of British Columbia, Vancouver, British Columbia, Canada
8) National Institute for Health Research, University of Southampton, Southampton, UK
9) Department of Neurosurgery, Sina Trauma and Surgery Research Center, Tehran University of Medical Sciences, Tehran, Iran.
10) University Spine Center, Balgrist University Hospital, Zurich, Switzerland.

For peer review only

11) Department of Neurosurgery, Medical College of Wisconsin, Milwaukee, Wisconsin, USA
12) Division of Neurosurgery, Thomas Jefferson University Hospital, Philadelphia, Pennsylvania, USA
13) Department of Neurosurgery and The Miami Project to Cure Paralysis, The Miller School of Medicine, University of Miami, Miami, FL, United States.

Key words:

Cervical, Myelopathy, OPLL, Spondylosis, Disc Herniation, Cervical Stenosis, protocol, outcome, dataset, Core outcomes in effectiveness trials (COMET), James Lind Alliance (JLA), research priorities, Delphi, consensus, audit, surveillance, common data elements (CDE)

Running title:

Evaluation of Knowledge Translation of Surgery for Degenerative Cervical Myelopathy

Abstract word count: 151

Text word count: 4845

Number of references: 45

Number of figures/tables: 5

Correspondence to:
Dr Benjamin Davies
Division of Neurosurgery
Department of Clinical Neurosciences
University of Cambridge
bd375@cam.ac.uk
Telephone: +44 1223 763366
Fax: +44 1223 763350

For peer review only

Acknowledgements

The research priorities were organized and funded by AO Spine through the AO Spine Knowledge Forum Spinal Cord Injury, a focused group of international Spinal Cord Injury experts. AO Spine is a clinical division of the AO Foundation, which is an independent medically-guided not-for-profit organization. Study support was provided directly through the AO Spine Research Department.

This research aligns with the AO Spine RECODE-DCM James Lind Alliance top research priority Raising Awareness selected by people living and working with DCM. For further information on how this process was conducted, why this question was prioritised, and global updates on currently aligned research, please visit aospine.org/recode/raising-awareness.

We thank Dr Joanna Crocker (Interdisciplinary Research in Health Sciences, University of Oxford) for her help with our methodology and statistical reporting.

AM is funded by a Clinician Scientist Fellowship (NIHR-CS-2017-17-010) from the UK National Institute for Health Research (NIHR) and supported by the NIHR Biomedical Research Centre at the University Hospitals Bristol NHS Foundation Trust and the University of Bristol. MRNK is supported by a NIHR Clinician Scientist Award, CS-2015-15-023. BMD is supported by a research fellowship from the Royal College of Surgeons (London), and the NIHR, United Kingdom. The views expressed in this publication are those of the authors and not necessarily those of the NHS, the National Institute for Health Research or the Department of Health.

Abstract

Objective

To explore whether a James Lind Alliance Priority Setting Partnership could provide insights on knowledge translation within the field of degenerative cervical myelopathy.

Design

Secondary analysis of a James Lind Alliance Priority Setting Partnership process for DCM (AO Spine RECODE DCM).

Methods

Research suggestions submitted by stakeholders but considered answered were identified. Sampling characteristics of respondents were also compared to the overall cohort to identify subgroups underserved by current knowledge translation.

Results

The survey was completed by 423 individuals from 68 different countries, including spinal surgeons, people with myelopathy, and other healthcare professionals. A total of 22% of participants submitted research suggestions that were considered 'answered'. Spinal surgeons were the group which was most likely to submit an 'answered' research question. Respondents from Asia were also more likely to submit 'answered' questions, when compared to other regions.

Conclusions

Knowledge translation challenges exist within the degenerative cervical myelopathy. This practical approach to measuring knowledge translation may offer a more responsive assessment to guide interventions, complementing existing metrics.

For peer review only

Introduction

Degenerative Cervical Myelopathy [DCM] is the most common cause of spinal cord dysfunction worldwide, affecting up to 2% of adults[1,2]. It arises when arthritic and/or congenital changes in the cervical spine cause progressive damage and injury to the cervical spinal cord. Today, despite treatment, most patients with DCM will be left with some disability. Furthermore, a recent comparative study demonstrated that people with DCM have amongst the lowest quality of life scores of chronic diseases[3,4]. Consequently, urgent progress is required.

To facilitate this, AO Spine Research objectives and Common Data Elements for Degenerative Cervical Myelopathy [RECODE-DCM] (aospine.org/recode), a multi-stakeholder consensus process was undertaken. This process aimed to accelerate research progress by defining key pieces of information which can better help individual studies deliver changes in care. It combined a number of consensus initiatives, including a James Lind Alliance Priority Setting Partnership (PSP), to establish the top 10 research uncertainties[5–7].

Whilst an improvement in outcomes will require further scientific advance and clinical research, for individuals to benefit from any such progress, new knowledge must also transfer into clinical practice[8]. This transfer of knowledge, or knowledge translation [KT], is not straightforward[9], and has been reported to take well over a decade in some cases[10]. For people with DCM, effective knowledge translation could be considered as important as knowledge discovery. This would be reflected in their selection of 'raising awareness' as the number one research priority for DCM[11].

A variety of strategies and frameworks have been proposed to optimize the KT process[12,13], including the formation of clinical practice guidelines. However, commonly this process

For peer review only

requires active surveillance and iteration. To that end, approaches to measure knowledge uptake are important but less well defined.

The aim of a PSP (Priority Setting Partnership) is to identify the critical knowledge gaps. This starts by seeking research suggestions from both people who have and who treat a condition (e.g., DCM), across relevant healthcare disciplines. These submissions are then processed and consolidated into summary questions. Each summary question is evaluated against the current evidence base and is removed from the process if it is felt to have already been answered. The remaining questions are then taken forward to be prioritized[7]. These steps for AO Spine RECODE DCM has been previously described[7].

Here, we explored the concept that the questions submitted by individuals as being “ongoing research questions” but considered otherwise to have been answered, might highlight areas where knowledge translation was particularly lacking.

For peer review only

Method

Survey

The protocol for AO Spine RECODE-DCM is published in the Global Spine Journal[14]. In summary, DCM stakeholders were recruited to an internet survey hosted by Calibrium (California, USA). AO Spine RECODE-DCM identified three principal stakeholder groups to partake in this initiative: Spinal Surgeons, Persons with DCM [PwCM] and their family or friends, and other healthcare professionals [oHCPs], including neurologists and physiotherapists.

The survey was advertised through national organizations, research networks and corresponding authors of DCM research. A detailed summary of the dissemination process has been published[6]. The survey was closed at the point of information saturation, defined as no additional unique research suggestions at a two-week interval. Following closure of the survey, research suggestions were processed by an information specialist[7,14]. Suggestions were grouped into common themes which were then used to form representative summary questions. All summary questions underwent an evidence checking process, including search of the literature and discussion with the Steering Committee. Questions were defined as either 'unanswered' or 'answered' depending on whether there was sufficient quality of evidence available in the literature. Questions that were considered 'answered' were removed from the process following review and discussion with the Steering Committee, composed of 6 neurosurgeons, 1 orthopedic surgeon, 2 neurologists, 1 primary care physician, 3 rehabilitation specialists and 12 PwCM[7].

Of the 76 summary questions generated, two were considered to have been answered: (1) *What is the safety and efficacy of surgical interventions for DCM?* and (2) *What is the efficacy and safety of anterior versus posterior surgery in patients with DCM?* The decision to remove this latter question also considered that Cervical Spondylotic Myelopathy Surgical

For peer review only

Trial (CSM-S, NCT02076113), a randomized controlled trial of anterior vs. posterior surgery, was in process. For brevity these will be referred to as the 'Effectiveness' and 'Anterior vs. Posterior' questions.

Analysis

Demographics of participants who submitted 'answered' and 'unanswered' summary questions were aggregated for analysis. For healthcare professionals, this included specialty, experience with DCM, age and country of employment. For PwCM or their supporters, this included country of residence and years lived with DCM. All participants were asked to provide their age and biological sex.

Geography is often an important consideration for knowledge translation for many reasons, including language, applicability to local practice and the physical barrier it can create for information exchange[15]. To explore this, country of residence or practice were aggregated into common zones – either by country if there was sufficient representation or continent if not. Countries were further categorized as Higher Income Countries or not, using the World Bank (worldbank.org) classification [22nd October, 2020]. In addition, we and others have identified that DCM research is largely derived from two geographical clusters: North America (Canada and the USA) and East Asia (Japan, Korea and China)[15,16]. To explore a relationship between research activity and knowledge translation, participants were also defined by whether they reside or practice within a research cluster or not.

Comparisons between groups, based on factors such as region and level of experience, were made using Chi-Squared test for categorical or ordinal data, and Mann Whitney U test for continuous data. Significance was defined as $p<0.05$.

Analysis and data visualisation were performed using R (v4.0.5; R Core Team, 2020) and RStudio (v1.4.1106; RStudio Team, 2021).

For peer review only

Patient and public involvement

Patient and carer representatives were engaged throughout the process. They helped define the scope and were involved in the review of all patient-facing media. They were involved in all steering group meetings and decisions. They collaborated with patient organisations and helped to reach a diverse range of patient and carers groups for the surveys and final workshop. Patient representatives will help disseminate the PSP findings and work with patient and charitable organisations to develop discrete research questions from the final priorities to take forward for funding.

For peer review only

Results

Summary

The survey was completed by 423 individuals from 68 different countries[6]. This included 232 surgeons (55%), 94 PwCM (22%) and 95 oHCP (23%). PwCM were principally from USA (41%) or the UK (32%). Surgeons and oHCP were more evenly distributed (Figure 1). In total, 95 (22%) participants submitted a research suggestion that mapped to one or both of these answered research questions; 51 (12%) 'Effective' and 44 (10%) 'Anterior vs Posterior'. This included 75 (32%) spinal surgeons, 12 (13%) PwCM and 8 (8%) oHCPs.

Submission of Research Suggestions that were "Answered" vs "Unanswered"

In the group that submitted a research suggestion that was deemed to be "answered" (i.e., around surgical "effectiveness" and "anterior vs posterior" surgery), there were 75 (79%) Surgeons, 12 (13%) oHCP and 8 (8%) PwCM. Spinal surgeons (p<0.005) and those of male sex (p<0.005) were more likely to submit a research suggestion that was already answered (Table 1; Supplementary Material 1).

*Table 1: Spinal Surgeon Stakeholders (N=232), Subgroup analysis. A high activity DCM research cluster was defined from a prior co-author network analysis – specifically DCM research activity clusters geographically to North America and East Asia (Japan, China and South Korea). *Significance, p<0.05.*

		Unanswered (%)		Answered (%)		P
Age		44.4		44.5		0.6
Male Gender		152	97%	73	97%	1
Region						0.21
	USA	13	8%	9	12%	
	UK	11	7%	7	9%	
	Canada	12	8%	2	3%	
	Europe	49	31%	18	24%	
	South America	6	4%	7	9%	

For peer review only

Middle East	3	2%	5	7%	
Asia	40	25%	16	21%	
Australasia	8	5%	5	7%	
Africa	15	10%	6	8%	
Research Cluster					
DCM Case Treated Yearly					0.78
0-25	28	18%	15	20%	
25-50	55	35%	24	32%	
50-100	47	30%	19	25%	
100+	27	17%	16	21%	
Year's Experience	13.6	9.7%	14.2	13.5%	
Neurosurgeon by Training	95	61%	45	60%	0.35
From a high-activity, DCM Research Cluster	43	27%	16	21%	0.41
High Income Country	111	71%	47	63%	0.28

Individuals were less likely to submit an answered research question if they resided or practiced within an active DCM research cluster (Japan, China, South Korea, USA or Canada) or high-income countries (Tables 1, 2, 3 and 4).

*Table 2: Comparison of respondent demographics of participants who submitted research suggestions that mapped to answered (N=95) compared to unanswered (N=328) summary questions. A high activity DCM research cluster was defined from a prior co-author network analysis – specifically DCM research activity clusters geographically to North America and East Asia (Japan, China and South Korea). *Significance, p<0.05.*

	**Unanswered (%)**		**Answered (%)**		**P**
N	328	78%	95	22%	
Stakeholder Group					*<0.005**
Spinal surgeons	157	48%	75	79%	

For peer review only

People with DCM and their supporters	82	25%	12	13%	
Other healthcare professionals	89	27%	8	8%	
Age (SD)	47.9	11.7	46.4	11.68	0.25
Male Gender	230	70%	84	88%	*<0.005*
Region					0.4
USA	62	19%	14	15%	
UK	43	13%	13	14%	
Canada	27	8%	4	4%	
Europe	82	25%	21	22%	
South America	14	4%	8	8%	
Middle East	10	3%	5	5%	
Asia	49	15%	18	19%	
Australasia	13	4%	6	6%	
Africa	28	9%	6	6%	
From a high-activity, DCM Research Cluster	109	33%	23	24%	0.12
High Income Country	250	76%	63	66%	0.07

*Table 1: Other Healthcare Professional Stakeholders (N=95), Subgroup Analysis. A high activity DCM research cluster was defined from a prior co-author network analysis – specifically DCM research activity clusters geographically to North America and East Asia (Japan, China and South Korea). *Significance, p<0.05.*

	**Unanswered (%)**		**Answered (%)**		**P**
Age	45.7				
Male Gender	52	58%	3	38%	0.4
Region					
USA	17	11%	0	0%	0.49

For peer review only

UK	7	4%	2	3%	
Canada	14	9%	2	3%	
Europe	28	18%	2	3%	
South America	3	2%	0	0%	
Middle East	5	3%	0	0%	
Asia	6	4%	1	1%	
Australasia	3	2%	1	1%	
Africa	6	4%	0	0%	
Research Cluster (Japan / China / N.America)					
DCM Volume					0.23
0-25	49	31%	7	9%	
25-50	20	13%	0	0%	
50-100	12	8%	0	0%	
100+	7	4%	1	1%	
Years Experience	14.7	9.7	9.9	13.5	0.88
From a high-activity, DCM Research Cluster	32	36%	2	25%	0.8
High Income Country	72	81%	6	75%	1
Discipline					
Neurologist	18	11%	0	0%	0.23
Physiotherapist	10	6%	1	1%	
Rehabilitation Medicine	9	6%	3	4%	
General Practitioner	9	6%	0	0%	
General Physician	10	6%	1	1%	
Other	32	20%	3	4%	

For peer review only

*Table 2: Persons with DCM or their supporters (friends or family), Subgroup Analysis. A high activity DCM research cluster was defined from a prior co-author network analysis – specifically DCM research activity clusters geographically to North America and East Asia (Japan, China and South Korea). *Significance, p<0.05.*

	Unanswered (%)		Answered (%)		P
Age	45.7		42.75		0.47
Male Gender	52	63%	3	38%	0.03
Region					
USA	32	39%	5	43%	0.96
UK	25	30%	4	33%	
Canada	1	1%	0	1%	
Europe	5	6%	1	7%	
South America	5	6%	1	7%	
Middle East	2	2%	0	3%	
Asia	3	4%	1	4%	
Australasia	2	2%	0	3%	
Africa	7	9%	0	9%	
Years Lived with DCM	5.5	4.8	5.3	4.2	0.8
From a high-activity, DCM Research Cluster	34	41%	10	45%	1
High Income Country	67	82%	10	89%	1

For peer review only

Professional experience or discipline was not associated with the likelihood of submitting an answered research question. Of note, no neurologist (N=18) submitted a research suggestion that mapped to an "answered" research question (Table 3).

Demographics were compared of those who submitted answered research suggestions, by whether it mapped to the 'Effectiveness' or to the 'Anterior vs. Posterior' questions. Spinal

surgeons and respondents from Asia ($p<0.05$) were more likely to submit research questions related to ‘Anterior vs. Posterior” approaches.

For peer review only

Discussion

Knowledge translation is a major issue in DCM. This is reflected by its selection as the number one research priority by AO Spine RECODE DCM – Raising Awareness[11]. This was also reflected within this analysis, as 22% of participants submitted research suggestions that were considered 'answered'. Spinal surgeons were more likely to submit an answered research question than oHCPs or PwCM. Anterior versus posterior surgery was more likely to be suggested by surgeons and respondents from Asia. Individuals living or practicing within a higher income country, or a country with high DCM research activity, tended to be less likely to submit an answered research suggestion; this association, however, was non-significant.

Can evidence checking of research suggestions act as a KT metric and inform KT strategy?

Ultimately, this was an exploratory analysis of an existing dataset, and cannot establish whether analysis of research suggestions is truly an effective KT metric. For example, many respondents in possession of the evidence may have considered the 'Anterior vs. Posterior' question to be unanswered. Our findings may instead reflect conflicting interpretations of the evidence, rather than poor KT. In this regard it was perhaps noteworthy that this question was more likely to be submitted by Asian surgeons where OPLL is more prevalent [32]. However, the results, taken in wider context, suggest promise.

Building on the significant growth in DCM research[17] and clinical evidence[18,19], clinical practice guidelines for DCM have been developed by AO Spine[20] and the World Federation of Neurosurgeons (WFNS) separately[21–24]. Whilst there remain many unanswered questions in DCM[14], these guidelines consolidate the current evidence on the effectiveness of surgical treatment[20]. Guidelines are considered one of the most effective tools for knowledge

For peer review only

translation [25,26]. Despite this, an audit of surgical practice has shown poor adherence to these guidelines[27], and DCM research continues to be dominated by investigations into these 'answered' research questions by surgeons[28]. This would suggest an ongoing KT gap and would align with the observed 75 (33%) surgeons who submitted at least one 'answered' question relating to this. This would also align with the on average 10-15 years[10] taken to bring new knowledge into routine practice.

Efforts to support the dissemination of these guidelines and inform evidence-based care are on-going. One of the challenges is the large number of specialities currently coordinating DCM care – all potential target audiences, e.g. general practice, neurology, physiotherapy, orthopedics, neurosurgery, rheumatology, gerontologists, and rehabilitation physicians[29]. Although the scope of answered questions was restricted to surgery, these research suggestions were still submitted by 8% of oHCP and 13% of PwCM; 8% and 13%.

The success of KT, or strategies to accelerate KT, are conventionally assessed through changes to guidelines, surveys of care providers and measurement of service/product/pathway adoption, where applicable[30]. Whilst valid, each of these metrics take considerable time to perform and, often, for example with respect to uptake within guidelines, would lag considerably a KT intervention. This means the recognition of successful or failed strategies, and/or need to iterate KT strategies, often is not very responsive.

Our experience here would suggest the assessment of the 'answered' research suggestions could offer a live snapshot of KT progress, concerning both patients and clinicians. Clearly its application may not be appropriate in all settings. For example, this analysis approach could not be applied to a PSP for perioperative care in Canada, where no research suggestions were deemed to be answered already[31]. This approach will also be vulnerable to selection bias. For example, as was the case in AO Spine RECODE DCM, engaging

For peer review only

stakeholders through electronic surveys outside of spinal surgery was very difficult. However, efforts could be taken to mitigate this.

Further, the relative differences in sampling characteristics may be helpful. In this study, participants were less likely to submit an answered research question if they came from a high-income country, or a country with higher DCM research activity. Questions relating to Anterior vs Posterior surgery were also more likely to come from Asia. Overall, these differences may indicate groups underserved by current KT strategies.

Factors contributing to Knowledge Translation gaps in DCM

There are several proposed barriers to rapid dissemination of DCM knowledge:

Terminology – First proposed in 2015[33], the umbrella term of DCM is still not universal[34]. 'Cervical spondylotic myelopathy' is the most commonly used term, but this has an inconsistent definition[34]. The use of variable terminology may therefore impede KT.

Geography/language – the major international guidelines have been published in English[20]. While this may not affect our study this affects international adoption of new knowledge[35].

Adaptation for local use - adaptation of knowledge to a local context is a key step in the knowledge-to-action cycle[36]. This is a pro-active process which must take place in individual hospitals and hospital networks[37].

There are also further barriers to the transfer of knowledge between different stakeholder groups.

For oHCPs, 'knowledge silos' have been described over the last decade between specialties[38,39]. The existence of different journals, vocabulary, professional organizations, and priorities are all believed to contribute to this[38,39]. Silos act to from closed-communication

For peer review only

loops which inhibit knowledge diffusion. We expect that this applies to surgeons, neurologists, and general practitioners in the case of DCM. Impaired collaboration also exists between clinicians and allied health professionals[40], which may form a barrier to KT within specialties.

KT to PwCM is also significant. If considering knowledge about surgical interventions, it has been long established that improved patient knowledge in the pre-operative phase enhances post-operative outcomes[41]. This is true in several domains, including post-operative compliance[42] and subjective pain reporting[43]. Barriers to the transfer of knowledge to PwCM include clinician knowledge and health literacy[44].

The relative comparison between 'answered' questions submitted by different stakeholder groups may reveal further insights about KT and the strategies used to tackle the above barriers. It is hoped the emergence of the RECODE-DCM community may also become a tool to address this[45].

Conclusion and Future Directions

Answered research questions were frequently submitted during the AO Spine RECODE DCM Priority Setting Partnership, indicating a knowledge translation problem in DCM. This practical approach to measuring knowledge translation may more widely offer a responsive assessment to guide interventions, complementing existing KT metrics which provide retrospective assessments. In the future, knowledge translation in AOSpine RECODE-DCM will need to involve considerable outreach to the broader community of health care providers involved with DCM, health care funders and policy makers and the public.

For peer review only

Ethics Statement

Ethical approval for the study was granted by the Human Biology Research Ethics Committee, University of Cambridge (HBREC.2019.14).

Contributors

BD, OM, DK, AM, LT, IS, ES, BA, BK, TG, VRM, CZ, PH, SK, JH, JW, JG, MF and MK were involved in the conceptualisation and design of this study. BD and JB wrote the first draft of the manuscript. BD, JB, OM, LT, BK and MF were involved in revising the manuscript. BD, JB, OM, DK, AM, LT, IS, ES, BA, BK, TG, VRM, CZ, PH, SK, JH, JW, JG, MF and MK reviewed and approved the final manuscript.

Competing interests

BD, JB, OM, IS, ES, MF and MK have voluntary roles at Myelopathy.org, an international DCM charity.

Funding

AM is funded by a Clinician Scientist Fellowship (NIHR-CS-2017-17-010) from the UK National Institute for Health Research (NIHR) and supported by the NIHR Biomedical Research Centre at the University Hospitals Bristol NHS Foundation Trust and the University of Bristol. MRNK is supported by a NIHR Clinician Scientist Award, CS-2015-15-023. BMD is supported by a research fellowship from the Royal College of Surgeons (London), and the NIHR, United Kingdom.

Disclaimer

The views expressed in this publication are those of the authors and not necessarily those of the NHS, the National Institute for Health Research or the Department of Health.

Data availability statement

For peer review only

Data are available upon reasonable request from the corresponding author.

For peer review only

References

1. Davies BM, Mowforth OD, Smith EK, Kotter MR. Degenerative cervical myelopathy. *BMJ*. 2018;360:k186. doi:10.1136/bmj.k186

2. Smith SS, Stewart ME, Davies BM, Kotter MRN. The Prevalence of Asymptomatic and Symptomatic Spinal Cord Compression on Magnetic Resonance Imaging: A Systematic Review and Meta-analysis. *Global Spine Journal*. 2021;11(4):597-607. doi:10.1177/2192568220934496

3. Pope DH, Mowforth OD, Davies BM, Kotter MRN. Diagnostic Delays Lead to Greater Disability in Degenerative Cervical Myelopathy and Represent a Health Inequality. *Spine (Phila Pa 1976)*. 2020;45(6):368-377. doi:10.1097/BRS.0000000000003305

4. Oh T, Lafage R, Lafage V, et al. Comparing Quality of Life in Cervical Spondylotic Myelopathy with Other Chronic Debilitating Diseases Using the Short Form Survey 36-Health Survey. *World Neurosurg*. 2017;106:699-706. doi:10.1016/j.wneu.2016.12.124

5. Cowan K. The James Lind alliance: tackling treatment uncertainties together. *J Ambul Care Manage*. 2010;33(3):241-248. doi:10.1097/JAC.0b013e3181e62cda

6. Mowforth OD, Khan DZ, Wong MY, et al. Gathering Global Perspectives to Establish the Research Priorities and Minimum Data Sets for Degenerative Cervical Myelopathy: Sampling Strategy of the First Round Consensus Surveys of AO Spine RECODE-DCM. *Global Spine J*. Published online December 8, 2021:21925682211047544. doi:10.1177/21925682211047546

7. Tetreault L, Mowforth O, Khan DZ, et al. James Lind Alliance Priority Setting Partnership for Degenerative Cervical Myelopathy [AO Spine RECODE-DCM]: An Overview of the Methodology Used to Process and Short-List Research Uncertainties. *Global Spine Journal*. 2022;12(1_suppl):19S-27S. doi:10.1177/21925682211062501

8. Djulbegovic B, Guyatt GH. Progress in evidence-based medicine: a quarter century on. *Lancet*. 2017;390(10092):415-423. doi:10.1016/S0140-6736(16)31592-6

9. Linskey ME. Evidence-Based Medicine for Neurosurgeons: Introduction and Methodology. *Guiding Neurosurgery by Evidence*. 2006;19:1-53. doi:10.1159/000095175

10. Morris ZS, Wooding S, Grant J. The answer is 17 years, what is the question: understanding time lags in translational research. *J R Soc Med*. 2011;104(12):510-520. doi:10.1258/jrsm.2011.110180

11. Davies BM. Improving awareness could transform outcomes in Degenerative Cervical Myelopathy (DCM) [AO Spine RECODE DCM Research Priority Number 1]. *Global Spine Journal*. Published online 2022.

12. Graham ID, Logan J, Harrison MB, et al. Lost in knowledge translation: time for a map? *J Contin Educ Health Prof*. 2006;26(1):13-24. doi:10.1002/chp.47

13. Grol R, Grimshaw J. From best evidence to best practice: effective implementation of change in patients' care. *The Lancet*. 2003;362(9391):1225-1230. doi:10.1016/S0140-6736(03)14546-1

For peer review only

14. Davies BM, Khan DZ, Mowforth OD, et al. RE-CODE DCM (REsearch Objectives and Common Data Elements for Degenerative Cervical Myelopathy): A Consensus Process to Improve Research Efficiency in DCM, Through Establishment of a Standardized Dataset for Clinical Research and the Definition of the Research Priorities. *Global Spine Journal*. 2019;9(1_suppl):65S-76S. doi:10.1177/2192568219832855

15. Duan Y, Nie W, Coakes E. Identifying key factors affecting transnational knowledge transfer. Published online December 2010. doi:10.1016/j.im.2010.08.003

16. Grodzinski N, Grodzinski B, Davies BM. Can co-authorship networks be used to predict author research impact? A machine-learning based analysis within the field of degenerative cervical myelopathy research. *PLoS One*. 2021;16(9):e0256997. doi:10.1371/journal.pone.0256997

17. Mowforth OD, Davies BM, Goh S, O'Neill CP, Kotter MRN. Research Inefficiency in Degenerative Cervical Myelopathy: Findings of a Systematic Review on Research Activity Over the Past 20 Years. *Global Spine Journal*. 2020;10(4):476-485. doi:10.1177/2192568219847439

18. Gnanalingham KK, Davies BM, Balamurali G, Titoria P, Doyle P, Abou-Zeid A. Improving levels of evidence in studies published in spinal journals from 1983 to 2011. *British Journal of Neurosurgery*. 2013;27(2):152-155. doi:10.3109/02688697.2012.737957

19. Donnally CJ, Butler AJ, Rush AJ, Bondar KJ, Wang MY, Eismont FJ. The most influential publications in cervical myelopathy. *J Spine Surg*. 2018;4(4):770-779. doi:10.21037/jss.2018.09.08

20. Fehlings MG, Tetreault LA, Riew KD, et al. A Clinical Practice Guideline for the Management of Patients With Degenerative Cervical Myelopathy: Recommendations for Patients With Mild, Moderate, and Severe Disease and Nonmyelopathic Patients With Evidence of Cord Compression. *Global Spine J*. 2017;7(3 Suppl):70S-83S. doi:10.1177/2192568217701914

21. Deora H, Kim SH, Behari S, et al. Anterior Surgical Techniques for Cervical Spondylotic Myelopathy: WFNS Spine Committee Recommendations. *Neurospine*. 2019;16(3):408-420. doi:10.14245/ns.1938250.125

22. Zileli M. Recommendations of WFNS Spine Committee. *Neurospine*. 2019;16(3):383-385. doi:10.14245/ns.19int003

23. Zileli M, Borkar SA, Sinha S, et al. Cervical Spondylotic Myelopathy: Natural Course and the Value of Diagnostic Techniques -WFNS Spine Committee Recommendations. *Neurospine*. 2019;16(3):386-402. doi:10.14245/ns.1938240.120

24. Bajamal AH, Kim SH, Arifianto MR, et al. Posterior Surgical Techniques for Cervical Spondylotic Myelopathy: WFNS Spine Committee Recommendations. *Neurospine*. 2019;16(3):421-434. doi:10.14245/ns.1938274.137

25. Newson R, King L, Rychetnik L, et al. A mixed methods study of the factors that influence whether intervention research has policy and practice impacts: perceptions of Australian researchers. *BMJ Open*. 2015;5(7):e008153. doi:10.1136/bmjopen-2015-008153

For peer review only

26. Tetreault L, Nater A, Garwood P, Badhiwala JH, Wilson JR, Fehlings MG. Development and Implementation of Clinical Practice Guidelines: An Update and Synthesis of the Literature With a Focus in Application to Spinal Conditions. *Global Spine Journal*. 2019;9(1_suppl):53S-64S. doi:10.1177/2192568219831689

27. Hilton B, Tempest-Mitchell J, Davies BM, et al. Cord compression defined by MRI is the driving factor behind the decision to operate in Degenerative Cervical Myelopathy despite poor correlation with disease severity. *PLoS One*. 2019;14(12):e0226020. doi:10.1371/journal.pone.0226020

28. Grodzinski B, Bestwick H, Bhatti F, et al. Research activity amongst DCM research priorities. *Acta Neurochir (Wien)*. 2021;163(6):1561-1568. doi:10.1007/s00701-021-04767-6

29. Hilton B, Tempest-Mitchell J, Davies B, Kotter M. Route to diagnosis of degenerative cervical myelopathy in a UK healthcare system: a retrospective cohort study. *BMJ Open*. 2019;9(5):e027000. doi:10.1136/bmjopen-2018-027000

30. Straus SE, Tetroe J, Graham I. Defining knowledge translation. *CMAJ*. 2009;181(3-4):165-168. doi:10.1503/cmaj.081229

31. McKeen DM, Banfield JC, McIsaac DI, et al. Top ten priorities for anesthesia and perioperative research: a report from the Canadian Anesthesia Research Priority Setting Partnership. *Can J Anaesth*. 2020;67(6):641-654. doi:10.1007/s12630-020-01607-6

32. Choi BW, Song KJ, Chang H. Ossification of the Posterior Longitudinal Ligament: A Review of Literature. *Asian Spine J*. 2011;5(4):267-276. doi:10.4184/asj.2011.5.4.267

33. Nouri A, Tetreault L, Singh A, Karadimas SK, Fehlings MG. Degenerative Cervical Myelopathy: Epidemiology, Genetics, and Pathogenesis. *Spine (Phila Pa 1976)*. 2015;40(12):E675-693. doi:10.1097/BRS.0000000000000913

34. Nouri A, Cheng JS, Davies B, Kotter M, Schaller K, Tessitore E. Degenerative Cervical Myelopathy: A Brief Review of Past Perspectives, Present Developments, and Future Directions. *J Clin Med*. 2020;9(2):535. doi:10.3390/jcm9020535

35. Amano T, González-Varo JP, Sutherland WJ. Languages Are Still a Major Barrier to Global Science. *PLOS Biology*. 2016;14(12):e2000933. doi:10.1371/journal.pbio.2000933

36. Straus SE, Tetroe JM, Graham ID. Knowledge translation is the use of knowledge in health care decision making. *J Clin Epidemiol*. 2011;64(1):6-10. doi:10.1016/j.jclinepi.2009.08.016

37. Harrison MB, Légaré F, Graham ID, Fervers B. Adapting clinical practice guidelines to local context and assessing barriers to their use. *CMAJ*. 2010;182(2):E78-E84. doi:10.1503/cmaj.081232

38. Harrigan MR, Jansen JO, Schnurman Z. Knowledge silos in pediatric blunt cerebrovascular injury. *Journal of Neurosurgery: Pediatrics*. 2020;27(2):239-240. doi:10.3171/2020.8.PEDS20636

For peer review only

39. Schnurman Z, Golfinos JG, Roland JT, Kondziolka D. Knowledge silos: assessing knowledge sharing between specialties through the vestibular schwannoma literature. *Journal of Neurosurgery*. 2017;129(5):1278-1285. doi:10.3171/2017.6.JNS171182

40. Zwarenstein M, Reeves S. Working together but apart: barriers and routes to nurse--physician collaboration. *Jt Comm J Qual Improv*. 2002;28(5):242-247, 209. doi:10.1016/s1070-3241(02)28024-4

41. Shuldham C. A review of the impact of pre-operative education on recovery from surgery. *Int J Nurs Stud*. 1999;36(2):171-177. doi:10.1016/s0020-7489(99)00010-3

42. Wong J, Wong S. A randomized controlled trial of a new approach to preoperative teaching and patient compliance. *International Journal of Nursing Studies*. 1985;22(2):105-115. doi:10.1016/0020-7489(85)90021-5

43. Devine EC. Effects of psychoeducational care for adult surgical patients: a meta-analysis of 191 studies. *Patient Educ Couns*. 1992;19(2):129-142. doi:10.1016/0738-3991(92)90193-m

44. Safeer RS, Keenan J. Health Literacy: The Gap Between Physicians and Patients. *AFP*. 2005;72(3):463-468.

45. Khan DZ, Hazenbiller O, Gronlund T, et al. The AO Spine RECODE-DCM International Collaborative—Establishing the Foundations for Accelerated and Patient-Centered Innovation. *Global Spine J*. 2022;12(1 Suppl):159S-171S. doi:10.1177/21925682211035381

For peer review only

Figure Captions

Figure 1: Number of stakeholders that submitted questions, by region of stakeholder. Spinal surgeons were mostly based in Europe and Asia, while PwCM were much more likely to be from the UK or USA.

For peer review only

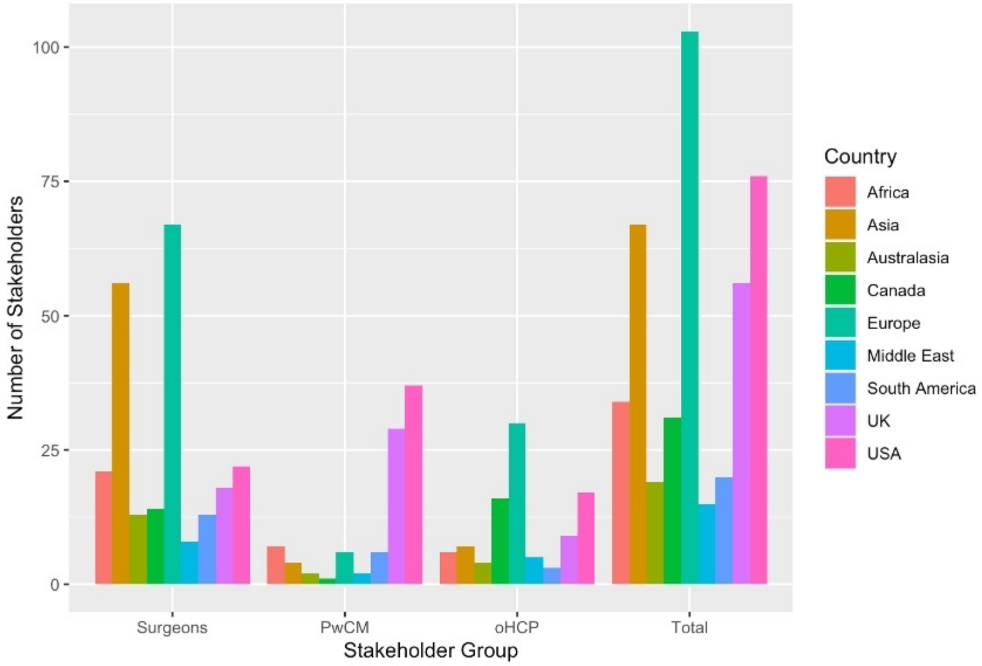

Figure 1: Number of stakeholders that submitted questions, by region of stakeholder. Spinal surgeons were mostly based in Europe and Asia, while PwCM were much more likely to be from the UK or USA.

150x102mm (144 x 144 DPI)

Supplementary Materials

Supplementary Material 1: Proportion of respondents of male sex submitting answered (Grey) and unanswered research questions (Orange), by principal stakeholder group and overall (black and red). Whilst male PwCM or their supporters were less likely to submit an answered research question, overall this association changed. It is likely this was driven by an interaction with Spinal Surgeons, who were almost universally male.

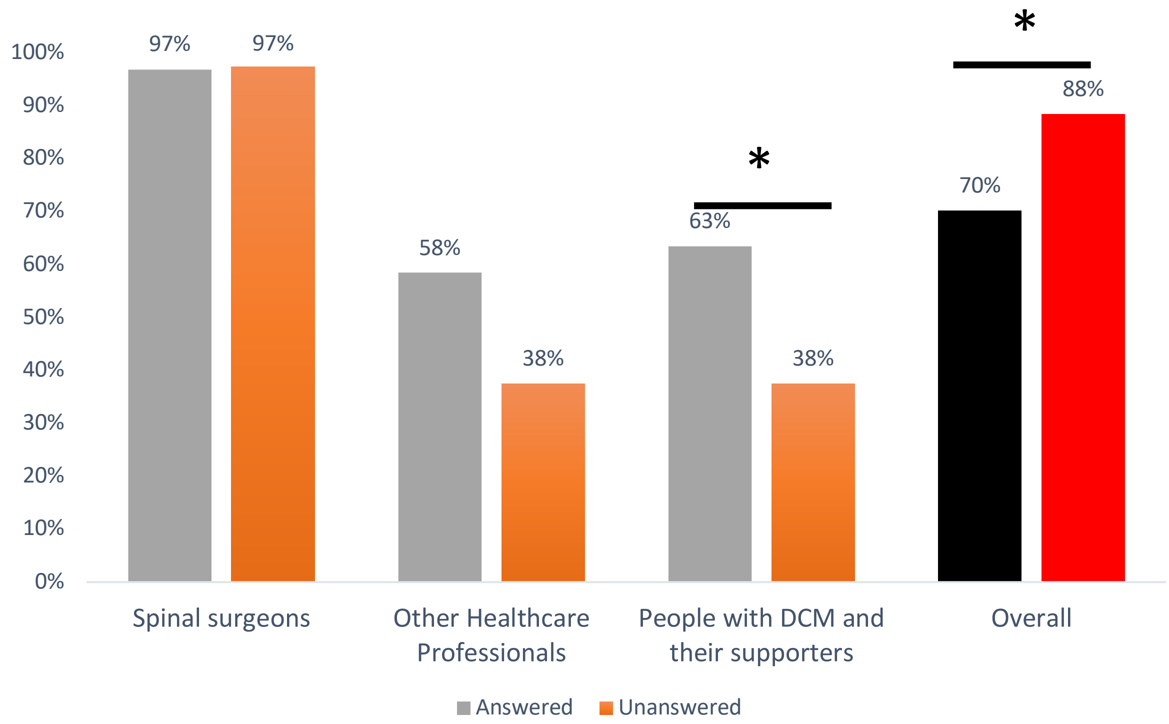

For peer review only - http://bmjopen.bmj.com/site/about/guidelines.xhtml

For peer review only

Secondary analysis of a James Lind Alliance priority setting partnership to facilitate knowledge translation in degenerative cervical myelopathy (DCM): Insights from AO Spine RECODE-DCM

Journal:	*BMJ Open*
Manuscript ID	bmjopen-2022-064296.R1
Article Type:	Original research
Date Submitted by the Author:	16-Dec-2022
Complete List of Authors:	Davies, Benjamin; University of Cambridge Neurosurgery, Department of Clinical Neurosurgery; University of Cambridge, University of Cambridge<br>Brannigan , Jamie ; University of Cambridge School of Clinical Medicine,<br>Mowforth, Oliver; University of Cambridge, Department of Academic Neurosurgery<br>Khan, Danyal; University College London,<br>McNair, Angus; University of Bristol, Centre for Surgical Research, Bristol Medical School: Population Health Sciences; North Bristol NHS Trust, GI Surgery<br>Tetreault, Lindsay; University of Toronto, Department of Surgery; University College Cork, Department of Medicine<br>Sadler, Iwan; Myelopathy.org<br>Sarewitz, Ellen; Myelopathy.org<br>Aarabi, Bizhan; University of Maryland Baltimore, Division of Neurosurgery,<br>Kwon, Brian; University of British Columbia, Division of Spine Surgery, Vancouver General Hospital<br>Gronlund, Toto ; National Institute for Health Research, University of Southampton, Southampton, UK<br>Rahimi-Movaghar, Vafa; Sina Trauma and Surgery Research Center, Academic Department of Neurological Surgery<br>Zipser, Carl Moritz; University Hospital Balgrist, Neurology<br>Hutchinson, Peter; University of Cambridge, Academic Neurosurgery<br>Kurpad, Shekar; Medical College of Wisconsin, Division of Neurosurgery<br>Harrop, James; Jefferson Health System,<br>Wilson, Jefferson R.; University of Toronto<br>Guest, James; University of Miami Miller School of Medicine, Department of Neurological Surgery<br>Fehlings, Michael G.; Toronto Western Hospital,<br>Kotter, Mark R.N.; University of Cambridge, Department of Clinical Neurosurgery; University of Cambridge
**Primary Subject Heading**:	Evidence based practice
Secondary Subject Heading:	Neurology, Surgery, Medical education and training, Qualitative research
Keywords:	NEUROSURGERY, Spine < ORTHOPAEDIC & TRAUMA SURGERY,

	SURGERY

SCHOLARONE™
Manuscripts

I, the Submitting Author has the right to grant and does grant on behalf of all authors of the Work (as defined in the below author licence), an exclusive licence and/or a non-exclusive licence for contributions from authors who are: i) UK Crown employees; ii) where BMJ has agreed a CC-BY licence shall apply, and/or iii) in accordance with the terms applicable for US Federal Government officers or employees acting as part of their official duties; on a worldwide, perpetual, irrevocable, royalty-free basis to BMJ Publishing Group Ltd ("BMJ") its licensees and where the relevant Journal is co-owned by BMJ to the co-owners of the Journal, to publish the Work in this journal and any other BMJ products and to exploit all rights, as set out in our licence.

The Submitting Author accepts and understands that any supply made under these terms is made by BMJ to the Submitting Author unless you are acting as an employee on behalf of your employer or a postgraduate student of an affiliated institution which is paying any applicable article publishing charge ("APC") for Open Access articles. Where the Submitting Author wishes to make the Work available on an Open Access basis (and intends to pay the relevant APC), the terms of reuse of such Open Access shall be governed by a Creative Commons licence – details of these licences and which Creative Commons licence will apply to this Work are set out in our licence referred to above.

Other than as permitted in any relevant BMJ Author's Self Archiving Policies, I confirm this Work has not been accepted for publication elsewhere, is not being considered for publication elsewhere and does not duplicate material already published. I confirm all authors consent to publication of this Work and authorise the granting of this licence.

For peer review only

Secondary analysis of a James Lind Alliance priority setting partnership to facilitate knowledge translation in degenerative cervical myelopathy (DCM): Insights from AO Spine RECODE-DCM

Benjamin M. Davies M.R.C.S, B.Sc. [1], Jamie F.M. Brannigan B.A.(Hons).[1], Oliver D. Mowforth B.A.(Hons). M.B. BChir. [1], Danyal Z. Khan MB, BCh, BAO [1], Angus G.K. McNair, F.R.C.S Ph.D FHEA[2], Lindsay Tetreault Ph.D.[3], Iwan Sadler [4], Ellen Sarewitz M.A [5], Bizhan Aarabi MD [6], Brian K Kwon M.D. Ph.D F.R.C.S.C [7], Toto Gronlund BSc, MSc [8], Vafa Rahimi-Movaghar MD[9], Carl Moritz Zipser MD FEBN[10], Peter J.A. Hutchinson Ph.D F.R.C.S FMedSci[1], Shekar N Kurpad M.D Ph.D[11], James Harrop M.D. [12], Jefferson R Wilson M.D. Ph.D F.R.C.S.C [3], James D. Guest, MD PhD FACS [13], Michael G. Fehlings M.D. Ph.D F.R.C.S.C [3], Mark R.N. Kotter M.D. M.Phil. Ph.D. [1]

1) Academic Neurosurgery Unit, Department of Clinical Neurosurgery, University of Cambridge, Cambridge, UK
2) Center for Surgical Research, Bristol Medical School: Population Health Sciences, University of Bristol, UK
3) Division of Neurosurgery and Spine Program, Department of Surgery, University of Toronto, Toronto, Ontario, Canada
4) Myelopathy.org, Cambridge, United Kingdom. (Registered Charity England and Wales, No 1178673)
5) Person with DCM and Director of Goffin Consultancy, Healthcare Consultants.
6) Division of Neurosurgery, Shock Trauma, University of Maryland, Baltimore, Maryland, USA
7) Division of Spine Surgery, Vancouver General Hospital, University of British Columbia, Vancouver, British Columbia, Canada
8) National Institute for Health Research, University of Southampton, Southampton, UK
9) Department of Neurosurgery, Sina Trauma and Surgery Research Center, Tehran University of Medical Sciences, Tehran, Iran.
10) University Spine Center, Balgrist University Hospital, Zurich, Switzerland.

For peer review only

11) Department of Neurosurgery, Medical College of Wisconsin, Milwaukee, Wisconsin, USA
12) Division of Neurosurgery, Thomas Jefferson University Hospital, Philadelphia, Pennsylvania, USA
13) Department of Neurosurgery and The Miami Project to Cure Paralysis, The Miller School of Medicine, University of Miami, Miami, FL, United States.

Key words:

Cervical, Myelopathy, OPLL, Spondylosis, Disc Herniation, Cervical Stenosis, protocol, outcome, dataset, Core outcomes in effectiveness trials (COMET), James Lind Alliance (JLA), research priorities, Delphi, consensus, audit, surveillance, common data elements (CDE)

Running title:

Evaluation of Knowledge Translation of Surgery for Degenerative Cervical Myelopathy

Abstract word count: 171

Text word count: 3320

Number of references: 47

Number of figures/tables: 5

Correspondence to:
Dr Benjamin Davies
Division of Neurosurgery
Department of Clinical Neurosciences
University of Cambridge
bd375@cam.ac.uk
Telephone: +44 1223 763366
Fax: +44 1223 763350

For peer review only

Abstract

<u>Objective</u>

To explore whether a James Lind Alliance Priority Setting Partnership could provide insights on knowledge translation within the field of degenerative cervical myelopathy (DCM).

<u>Design</u>

Secondary analysis of a James Lind Alliance Priority Setting Partnership process for DCM.

<u>Sample and setting</u>

DCM stake holders, including spinal surgeons, people with myelopathy, and other healthcare professionals were surveyed internationally.

<u>Methods</u>

Research suggestions submitted by stakeholders but considered answered were identified. Sampling characteristics of respondents were compared to the overall cohort to identify subgroups underserved by current knowledge translation.

<u>Results</u>

The survey was completed by 423 individuals from 68 different countries. A total of 22% of participants submitted research suggestions that were considered ‘answered’. Spinal surgeons were the group which was most likely to submit an ‘answered’ research question. Respondents from Asia were also more likely to submit ‘answered’ questions, when compared to other regions.

<u>Conclusions</u>

Knowledge translation challenges exist within degenerative cervical myelopathy. This practical approach to measuring knowledge translation may offer a more responsive assessment to guide interventions, complementing existing metrics.

For peer review only

Strengths and limitations of this study

- A large number of stakeholders including patients, clinicians, and researchers were surveyed, generating 76 research questions.
- Responses came from individuals in 68 countries,
- The protocol for collecting the data analysed in this study has been published previously.
- The dissemination of the online survey through national organizations and research networks makes this study vulnerable to response bias.

For peer review only

Introduction

Degenerative Cervical Myelopathy [DCM] is the most common cause of spinal cord dysfunction worldwide, affecting up to 2% of adults[1,2]. It arises when arthritic and/or congenital changes in the cervical spine cause progressive damage and injury to the cervical spinal cord. Today, despite treatment, most patients with DCM will be left with some disability. This is often due to missed or late diagnosis[3]. Furthermore, a recent comparative study demonstrated that people with DCM have amongst the lowest quality of life scores of chronic diseases[4,5]. Consequently, urgent progress is required.

To facilitate this, AO Spine Research objectives and Common Data Elements for Degenerative Cervical Myelopathy [RECODE-DCM] (aospine.org/recode), a multi-stakeholder consensus process was undertaken. This process aimed to accelerate research progress by defining key pieces of information which can better help individual studies deliver changes in care. It combined a number of consensus initiatives, including a James Lind Alliance Priority Setting Partnership (PSP), to establish the top 10 research uncertainties[6–8].

Whilst an improvement in outcomes will require further scientific advance and clinical research, for individuals to benefit from any such progress, new knowledge must also transfer into clinical practice[9]. This transfer of knowledge, or knowledge translation [KT], is not straightforward[10], and has been reported to take well over a decade in some cases[11]. For people with DCM, effective knowledge translation could be considered as important as knowledge discovery. This would be reflected in their selection of ‘raising awareness’ as the number one research priority for DCM[12].

A variety of strategies and frameworks have been proposed to optimize the KT process[13,14], including the formation of clinical practice guidelines. However, commonly this process

For peer review only

requires active surveillance and iteration. To that end, approaches to measure knowledge uptake are important but less well defined.

The aim of a PSP (Priority Setting Partnership) is to identify the critical knowledge gaps. This starts by seeking research suggestions from both people who have and who treat a condition (e.g., DCM), across relevant healthcare disciplines. These submissions are then processed and consolidated into summary questions. Each summary question is evaluated against the current evidence base and is removed from the process if it is felt to have already been answered. The remaining questions are then taken forward to be prioritized[8]. These steps for AO Spine RECODE DCM has been previously described[8].

Here, we explored the concept that the questions submitted by individuals as being "ongoing research questions" but considered otherwise to have been answered, might highlight areas where knowledge translation was particularly lacking.

For peer review only

Method

Survey

The protocol for AO Spine RECODE-DCM is published in the Global Spine Journal[15]. In summary, DCM stakeholders were recruited to an internet survey hosted by Calibrium (California, USA). AO Spine RECODE-DCM identified three principal stakeholder groups to partake in this initiative: Spinal Surgeons, Persons with DCM [PwCM] and their family or friends, and other healthcare professionals [oHCPs], including neurologists and physiotherapists.

The survey was advertised through national organizations, research networks and corresponding authors of DCM research. A detailed summary of the dissemination process has been published[7]. The survey was closed at the point of information saturation, defined as no additional unique research suggestions at a two-week interval. Following closure of the survey, research suggestions were processed by an information specialist[8,15]. Suggestions were grouped into common themes which were then used to form representative summary questions. All summary questions underwent an evidence checking process, including search of the literature and discussion with the Steering Committee. Questions were defined as either 'unanswered' or 'answered' depending on whether there was sufficient quality of evidence available in the literature. Scoping reviews of the literature were conducted by LT, the designated information specialist for this JLA PSP, to find evidence to support this process. Questions that were considered 'answered' were removed from the process following review and discussion with the Steering Committee, composed of 6 neurosurgeons, 1 orthopedic surgeon, 2 neurologists, 1 primary care physician, 3 rehabilitation specialists and 12 PwCM[8].

Of the 76 summary questions generated, two were considered to have been answered: (1) *What is the safety and efficacy of surgical interventions for DCM?* and (2) *What is the*

For peer review only

efficacy and safety of anterior versus posterior surgery in patients with DCM? The decision to remove this latter question also considered that Cervical Spondylotic Myelopathy Surgical Trial (CSM-S, NCT02076113), a randomized controlled trial of anterior vs. posterior surgery, was in process. For brevity these will be referred to as the 'Effectiveness' and 'Anterior vs. Posterior' questions. The remaining 74 questions, which were considered unanswered, are publicly available on the James Lind Alliance PSP website[16].

For peer review only

Analysis

Demographics of participants who submitted 'answered' and 'unanswered' summary questions were aggregated for analysis. For healthcare professionals, this included specialty, experience with DCM, age and country of employment. For PwCM or their supporters, this included country of residence and years lived with DCM. All participants were asked to provide their age and biological sex.

Geography is often an important consideration for knowledge translation for many reasons, including language, applicability to local practice and the physical barrier it can create for information exchange[17]. To explore this, country of residence or practice were aggregated into common zones – either by country if there was sufficient representation or continent if not. Countries were further categorized as Higher Income Countries or not, using the World Bank (worldbank.org) classification [22nd October, 2020]. In addition, we and others have identified that DCM research is largely derived from two geographical clusters: North America (Canada and the USA) and East Asia (Japan, Korea and China)[17,18]. To explore a relationship between research activity and knowledge translation, participants were also defined by whether they reside or practice within a research cluster or not.

Comparisons between groups, based on factors such as region and level of experience, were made using Chi-Squared test for categorical or ordinal data, and Mann Whitney U test for continuous data. Significance was defined as $p<0.05$.

Analysis and data visualisation were performed using R (v4.0.5; R Core Team, 2020) and RStudio (v1.4.1106; RStudio Team, 2021).

Patient and public involvement

Patient and carer representatives were engaged throughout the process. They helped define the scope and were involved in the review of all patient-facing media. They were involved in all steering group meetings and decisions. They collaborated with patient organisations and helped to reach a diverse range of patient and carers groups for the surveys and final workshop. Patient representatives will help disseminate the PSP findings and work with patient and charitable organisations to develop discrete research questions from the final priorities to take forward for funding.

For peer review only

Results

Summary

The survey was completed by 423 individuals from 68 different countries[7]. This included 232 surgeons (55%), 94 PwCM (22%) and 95 oHCP (23%). PwCM were principally from USA (41%) or the UK (32%). Surgeons and oHCP were more evenly distributed (Figure 1). In total, 95 (22%) participants submitted a research suggestion that mapped to one or both of these answered research questions; 51 (12%) 'Effective' and 44 (10%) 'Anterior vs Posterior'. This included 75 (32%) spinal surgeons, 12 (13%) PwCM and 8 (8%) oHCPs.

Submission of Research Suggestions that were "Answered" vs "Unanswered"

In the group that submitted a research suggestion that was deemed to be "answered" (i.e., around surgical "effectiveness" and "anterior vs posterior" surgery), there were 75 (79%) Surgeons, 12 (13%) oHCP and 8 (8%) PwCM. Spinal surgeons ($p<0.005$) and those of male sex ($p<0.005$) were more likely to submit a research suggestion that was already answered (Table 1; Supplementary Material 1).

*Table 1: Spinal Surgeon Stakeholders (N=232), Subgroup analysis. A high activity DCM research cluster was defined from a prior co-author network analysis – specifically DCM research activity clusters geographically to North America and East Asia (Japan, China and South Korea). *Significance, p<0.05.*

		Unanswered (%)		Answered (%)		P
Age		44.4		44.5		0.6
Male Gender		152	97%	73	97%	1
Region						0.21
	USA	13	8%	9	12%	
	UK	11	7%	7	9%	
	Canada	12	8%	2	3%	
	Europe	49	31%	18	24%	
	South America	6	4%	7	9%	

For peer review only

Middle East	3	2%	5	7%	
Asia	40	25%	16	21%	
Australasia	8	5%	5	7%	
Africa	15	10%	6	8%	
Research Cluster					
DCM Case Treated Yearly					0.78
0-25	28	18%	15	20%	
25-50	55	35%	24	32%	
50-100	47	30%	19	25%	
100+	27	17%	16	21%	
Year's Experience	13.6	9.7%	14.2	13.5%	
Neurosurgeon by Training	95	61%	45	60%	0.35
From a high-activity, DCM Research Cluster	43	27%	16	21%	0.41
High Income Country	111	71%	47	63%	0.28

Individuals were less likely to submit an answered research question if they resided or practiced within an active DCM research cluster (Japan, China, South Korea, USA or Canada) or high-income countries (Tables 1, 2, 3 and 4).

*Table 2: Comparison of respondent demographics of participants who submitted research suggestions that mapped to answered (N=95) compared to unanswered (N=328) summary questions. A high activity DCM research cluster was defined from a prior co-author network analysis – specifically DCM research activity clusters geographically to North America and East Asia (Japan, China and South Korea). *Significance, p<0.05.*

	**Unanswered (%)**		**Answered (%)**		**P**
N	328	78%	95	22%	
Stakeholder Group					*<0.005**
Spinal surgeons	157	48%	75	79%	

For peer review only

People with DCM and their supporters	82	25%	12	13%	
Other healthcare professionals	89	27%	8	8%	
Age (SD)	47.9	11.7	46.4	11.68	0.25
Male Gender	230	70%	84	88%	*<0.005*
Region					0.4
USA	62	19%	14	15%	
UK	43	13%	13	14%	
Canada	27	8%	4	4%	
Europe	82	25%	21	22%	
South America	14	4%	8	8%	
Middle East	10	3%	5	5%	
Asia	49	15%	18	19%	
Australasia	13	4%	6	6%	
Africa	28	9%	6	6%	
From a high-activity, DCM Research Cluster	109	33%	23	24%	0.12
High Income Country	250	76%	63	66%	0.07

*Table 1: Other Healthcare Professional Stakeholders (N=95), Subgroup Analysis. A high activity DCM research cluster was defined from a prior co-author network analysis – specifically DCM research activity clusters geographically to North America and East Asia (Japan, China and South Korea). *Significance, p<0.05.*

	Unanswered (%)		Answered (%)		P
Age	45.7		42.8		
Male Gender	52	58%	3	38%	0.4
Region					
USA	17	11%	0	0%	0.49

For peer review only

UK	7	4%	2	3%	
Canada	14	9%	2	3%	
Europe	28	18%	2	3%	
South America	3	2%	0	0%	
Middle East	5	3%	0	0%	
Asia	6	4%	1	1%	
Australasia	3	2%	1	1%	
Africa	6	4%	0	0%	
Research Cluster (Japan / China / N.America)					
DCM Volume					0.23
0-25	49	31%	7	9%	
25-50	20	13%	0	0%	
50-100	12	8%	0	0%	
100+	7	4%	1	1%	
Years Experience	14.7	9.7	9.9	13.5	0.88
From a high-activity, DCM Research Cluster	32	36%	2	25%	0.8
High Income Country	72	81%	6	75%	1
Discipline					
Neurologist	18	11%	0	0%	0.23
Physiotherapist	10	6%	1	1%	
Rehabilitation Medicine	9	6%	3	4%	
General Practitioner	9	6%	0	0%	
General Physician	10	6%	1	1%	
Other	32	20%	3	4%	

For peer review only

*Table 2: Persons with DCM or their supporters (friends or family), Subgroup Analysis. A high activity DCM research cluster was defined from a prior co-author network analysis – specifically DCM research activity clusters geographically to North America and East Asia (Japan, China and South Korea). *Significance, p<0.05.*

	Unanswered (%)		Answered (%)		P
Age	45.7		42.75		0.47
Male Gender	52	63%	3	38%	0.03
Region					
USA	32	39%	5	43%	0.96
UK	25	30%	4	33%	
Canada	1	1%	0	1%	
Europe	5	6%	1	7%	
South America	5	6%	1	7%	
Middle East	2	2%	0	3%	
Asia	3	4%	1	4%	
Australasia	2	2%	0	3%	
Africa	7	9%	0	9%	
Years Lived with DCM	5.5	4.8	5.3	4.2	0.8
From a high-activity, DCM Research Cluster	34	41%	10	45%	1
High Income Country	67	82%	10	89%	1

For peer review only

Professional experience or discipline was not associated with the likelihood of submitting an answered research question. Of note, no neurologist (N=18) submitted a research suggestion that mapped to an “answered” research question (Table 3).

Demographics were compared of those who submitted answered research suggestions, by whether it mapped to the ‘Effectiveness’ or to the ‘Anterior vs. Posterior’ questions. Spinal

surgeons and respondents from Asia ($p<0.05$) were more likely to submit research questions related to 'Anterior vs. Posterior" approaches.

For peer review only

Discussion

Knowledge translation is a major issue in DCM. This is reflected by its selection as the number one research priority by AO Spine RECODE DCM – Raising Awareness[12]. This was also reflected within this analysis, as 22% of participants submitted research suggestions that were considered 'answered'. Spinal surgeons were more likely to submit an answered research question than oHCPs or PwCM. Anterior versus posterior surgery was more likely to be suggested by surgeons and respondents from Asia. Individuals living or practicing within a higher income country, or a country with high DCM research activity, tended to be less likely to submit an answered research suggestion; this association, however, was non-significant.

<u>Can evidence checking of research suggestions act as a KT metric and inform KT strategy?</u>

Ultimately, this was an exploratory analysis of an existing dataset, and cannot establish whether analysis of research suggestions is truly an effective KT metric. For example, many respondents in possession of the evidence may have considered the 'Anterior vs. Posterior' question to be unanswered. Our findings may instead reflect conflicting interpretations of the evidence, rather than poor KT. In this regard it was perhaps noteworthy that this question was more likely to be submitted by Asian surgeons where OPLL is more prevalent [19]. However, the results, taken in wider context, suggest promise.

Building on the significant growth in DCM research[20] and clinical evidence[21,22], clinical practice guidelines for DCM have been developed by AO Spine[23] and the World Federation of Neurosurgeons (WFNS) separately[24–27]. Whilst there remain many unanswered questions in DCM[15], these guidelines consolidate the current evidence on the effectiveness of surgical treatment[23]. Guidelines are considered one of the most effective tools for knowledge

For peer review only

translation [28,29]. Despite this, an audit of surgical practice has shown poor adherence to these guidelines[30], and DCM research continues to be dominated by investigations into these 'answered' research questions by surgeons[31]. This would suggest an ongoing KT gap and would align with the observed 75 (33%) surgeons who submitted at least one 'answered' question relating to this. This would also align with the on average 10-15 years[11] taken to bring new knowledge into routine practice.

Efforts to support the dissemination of these guidelines and inform evidence-based care are on-going. One of the challenges is the large number of specialities currently coordinating DCM care – all potential target audiences, e.g. general practice, neurology, physiotherapy, orthopedics, neurosurgery, rheumatology, gerontologists, and rehabilitation physicians[32]. Although the scope of answered questions was restricted to surgery, these research suggestions were still submitted by 8% of oHCP and 13% of PwCM; 8% and 13%.

The success of KT, or strategies to accelerate KT, are conventionally assessed through changes to guidelines, surveys of care providers and measurement of service/product/pathway adoption, where applicable[33]. Whilst valid, each of these metrics take considerable time to perform and, often, for example with respect to uptake within guidelines, would lag considerably a KT intervention. This means the recognition of successful or failed strategies, and/or need to iterate KT strategies, often is not very responsive.

Our experience here would suggest the assessment of the 'answered' research suggestions could offer a live snapshot of KT progress, concerning both patients and clinicians. Clearly its application may not be appropriate in all settings. For example, this analysis approach could not be applied to a PSP for perioperative care in Canada, where no research suggestions were deemed to be answered already[34]. This approach will also be vulnerable to selection bias. For example, as was the case in AO Spine RECODE DCM, engaging

For peer review only

stakeholders through electronic surveys outside of spinal surgery was very difficult. However, efforts could be taken to mitigate this.

Further, the relative differences in sampling characteristics may be helpful. In this study, participants were less likely to submit an answered research question if they came from a high-income country, or a country with higher DCM research activity. Questions relating to Anterior vs Posterior surgery were also more likely to come from Asia. Overall, these differences may indicate groups underserved by current KT strategies.

Factors contributing to Knowledge Translation gaps in DCM

There are several proposed barriers to rapid dissemination of DCM knowledge:

Terminology – First proposed in 2015[35], the umbrella term of DCM is still not universal[36]. 'Cervical spondylotic myelopathy' is the most commonly used term, but this has an inconsistent definition[36]. The use of variable terminology may therefore impede KT.

Geography/language – the major international guidelines have been published in English[23]. While this may not affect our study this affects international adoption of new knowledge[37].

Adaptation for local use - adaptation of knowledge to a local context is a key step in the knowledge-to-action cycle[38]. This is a pro-active process which must take place in individual hospitals and hospital networks[39].

There are also further barriers to the transfer of knowledge between different stakeholder groups.

For oHCPs, 'knowledge silos' have been described over the last decade between specialties[40,41]. The existence of different journals, vocabulary, professional organizations, and priorities are all believed to contribute to this[40,41]. Silos act to from closed-communication

For peer review only

loops which inhibit knowledge diffusion. We expect that this applies to surgeons, neurologists, and general practitioners in the case of DCM. Impaired collaboration also exists between clinicians and allied health professionals[42], which may form a barrier to KT within specialties.

KT to PwCM is also significant. If considering knowledge about surgical interventions, it has been long established that improved patient knowledge in the pre-operative phase enhances post-operative outcomes[43]. This is true in several domains, including post-operative compliance[44] and subjective pain reporting[45]. Barriers to the transfer of knowledge to PwCM include clinician knowledge and health literacy[46].

The relative comparison between 'answered' questions submitted by different stakeholder groups may reveal further insights about KT and the strategies used to tackle the above barriers. It is hoped the emergence of the RECODE-DCM community may also become a tool to address this[47].

Conclusion and Future Directions

Answered research questions were frequently submitted during the AO Spine RECODE DCM Priority Setting Partnership, indicating a knowledge translation problem in DCM. This practical approach to measuring knowledge translation may more widely offer a responsive assessment to guide interventions, complementing existing KT metrics which provide retrospective assessments. In the future, knowledge translation in AOSpine RECODE-DCM will need to involve considerable outreach to the broader community of health care providers involved with DCM, health care funders and policy makers and the public.

For peer review only

Ethics Statement

Ethical approval for the study was granted by the Human Biology Research Ethics Committee, University of Cambridge (HBREC.2019.14).

Contributors

BD, OM, DK, AM, LT, IS, ES, BA, BK, TG, VRM, CZ, PH, SK, JH, JW, JG, MF and MK were involved in the conceptualisation and design of this study. BD and JB wrote the first draft of the manuscript. BD, JB, OM, LT, BK and MF were involved in revising the manuscript. BD, JB, OM, DK, AM, LT, IS, ES, BA, BK, TG, VRM, CZ, PH, SK, JH, JW, JG, MF and MK reviewed and approved the final manuscript.

Competing interests

BD, JB, OM, IS, ES, MF and MK have voluntary roles at Myelopathy.org, an international DCM charity.

Funding

AM is funded by a Clinician Scientist Fellowship (NIHR-CS-2017-17-010) from the UK National Institute for Health Research (NIHR) and supported by the NIHR Biomedical Research Centre at the University Hospitals Bristol NHS Foundation Trust and the University of Bristol. MRNK is supported by a NIHR Clinician Scientist Award, CS-2015-15-023. BMD is supported by a research fellowship from the Royal College of Surgeons (London), and the NIHR, United Kingdom.

Disclaimer

The views expressed in this publication are those of the authors and not necessarily those of the NHS, the National Institute for Health Research or the Department of Health.

For peer review only

Data availability statement

Data are available upon reasonable request from the corresponding author.

For peer review only

Acknowledgements

The research priorities were organized and funded by AO Spine through the AO Spine Knowledge Forum Spinal Cord Injury, a focused group of international Spinal Cord Injury experts. AO Spine is a clinical division of the AO Foundation, which is an independent medically-guided not-for-profit organization. Study support was provided directly through the AO Spine Research Department.

This research aligns with the AO Spine RECODE-DCM James Lind Alliance top research priority Raising Awareness selected by people living and working with DCM. For further information on how this process was conducted, why this question was prioritised, and global updates on currently aligned research, please visit aospine.org/recode/raising-awareness.

We thank Dr Joanna Crocker (Interdisciplinary Research in Health Sciences, University of Oxford) for her help with our methodology and statistical reporting.

AM is funded by a Clinician Scientist Fellowship (NIHR-CS-2017-17-010) from the UK National Institute for Health Research (NIHR) and supported by the NIHR Biomedical Research Centre at the University Hospitals Bristol NHS Foundation Trust and the University of Bristol. MRNK is supported by a NIHR Clinician Scientist Award, CS-2015-15-023. BMD is supported by a research fellowship from the Royal College of Surgeons (London), and the NIHR, United Kingdom. The views expressed in this publication are those of the authors and not necessarily those of the NHS, the National Institute for Health Research or the Department of Health.

For peer review only

References

1. Davies BM, Mowforth OD, Smith EK, Kotter MR. Degenerative cervical myelopathy. *BMJ*. 2018;360:k186. doi:10.1136/bmj.k186

2. Smith SS, Stewart ME, Davies BM, Kotter MRN. The Prevalence of Asymptomatic and Symptomatic Spinal Cord Compression on Magnetic Resonance Imaging: A Systematic Review and Meta-analysis. *Global Spine Journal*. 2021;11(4):597-607. doi:10.1177/2192568220934496

3. Grodzinski B, Stubbs DJ, Davies BM. Most degenerative cervical myelopathy remains undiagnosed, particularly amongst the elderly: modelling the prevalence of degenerative cervical myelopathy in the United Kingdom. *J Neurol*. Published online September 2, 2022. doi:10.1007/s00415-022-11349-8

4. Pope DH, Mowforth OD, Davies BM, Kotter MRN. Diagnostic Delays Lead to Greater Disability in Degenerative Cervical Myelopathy and Represent a Health Inequality. *Spine (Phila Pa 1976)*. 2020;45(6):368-377. doi:10.1097/BRS.0000000000003305

5. Oh T, Lafage R, Lafage V, et al. Comparing Quality of Life in Cervical Spondylotic Myelopathy with Other Chronic Debilitating Diseases Using the Short Form Survey 36-Health Survey. *World Neurosurg*. 2017;106:699-706. doi:10.1016/j.wneu.2016.12.124

6. Cowan K. The James Lind alliance: tackling treatment uncertainties together. *J Ambul Care Manage*. 2010;33(3):241-248. doi:10.1097/JAC.0b013e3181e62cda

7. Mowforth OD, Khan DZ, Wong MY, et al. Gathering Global Perspectives to Establish the Research Priorities and Minimum Data Sets for Degenerative Cervical Myelopathy: Sampling Strategy of the First Round Consensus Surveys of AO Spine RECODE-DCM. *Global Spine J*. Published online December 8, 2021:21925682211047544. doi:10.1177/21925682211047546

8. Tetreault L, Mowforth O, Khan DZ, et al. James Lind Alliance Priority Setting Partnership for Degenerative Cervical Myelopathy [AO Spine RECODE-DCM]: An Overview of the Methodology Used to Process and Short-List Research Uncertainties. *Global Spine Journal*. 2022;12(1_suppl):19S-27S. doi:10.1177/21925682211062501

9. Djulbegovic B, Guyatt GH. Progress in evidence-based medicine: a quarter century on. *Lancet*. 2017;390(10092):415-423. doi:10.1016/S0140-6736(16)31592-6

10. Linskey ME. Evidence-Based Medicine for Neurosurgeons: Introduction and Methodology. *Guiding Neurosurgery by Evidence*. 2006;19:1-53. doi:10.1159/000095175

11. Morris ZS, Wooding S, Grant J. The answer is 17 years, what is the question: understanding time lags in translational research. *J R Soc Med*. 2011;104(12):510-520. doi:10.1258/jrsm.2011.110180

12. Davies BM. Improving awareness could transform outcomes in Degenerative Cervical Myelopathy (DCM) [AO Spine RECODE DCM Research Priority Number 1]. *Global Spine Journal*. Published online 2022.

13. Graham ID, Logan J, Harrison MB, et al. Lost in knowledge translation: time for a map? *J Contin Educ Health Prof*. 2006;26(1):13-24. doi:10.1002/chp.47

For peer review only

14. Grol R, Grimshaw J. From best evidence to best practice: effective implementation of change in patients' care. *The Lancet*. 2003;362(9391):1225-1230. doi:10.1016/S0140-6736(03)14546-1

15. Davies BM, Khan DZ, Mowforth OD, et al. RE-CODE DCM (REsearch Objectives and Common Data Elements for Degenerative Cervical Myelopathy): A Consensus Process to Improve Research Efficiency in DCM, Through Establishment of a Standardized Dataset for Clinical Research and the Definition of the Research Priorities. *Global Spine Journal*. 2019;9(1_suppl):65S-76S. doi:10.1177/2192568219832855

16. Degenerative Cervical Myelopathy | James Lind Alliance. Accessed December 15, 2022. https://www.jla.nihr.ac.uk/priority-setting-partnerships/Degenerative-Cervical-Myelopathy/

17. Duan Y, Nie W, Coakes E. Identifying key factors affecting transnational knowledge transfer. Published online December 2010. doi:10.1016/j.im.2010.08.003

18. Grodzinski N, Grodzinski B, Davies BM. Can co-authorship networks be used to predict author research impact? A machine-learning based analysis within the field of degenerative cervical myelopathy research. *PLoS One*. 2021;16(9):e0256997. doi:10.1371/journal.pone.0256997

19. Choi BW, Song KJ, Chang H. Ossification of the Posterior Longitudinal Ligament: A Review of Literature. *Asian Spine J*. 2011;5(4):267-276. doi:10.4184/asj.2011.5.4.267

20. Mowforth OD, Davies BM, Goh S, O'Neill CP, Kotter MRN. Research Inefficiency in Degenerative Cervical Myelopathy: Findings of a Systematic Review on Research Activity Over the Past 20 Years. *Global Spine Journal*. 2020;10(4):476-485. doi:10.1177/2192568219847439

21. Gnanalingham KK, Davies BM, Balamurali G, Titoria P, Doyle P, Abou-Zeid A. Improving levels of evidence in studies published in spinal journals from 1983 to 2011. *British Journal of Neurosurgery*. 2013;27(2):152-155. doi:10.3109/02688697.2012.737957

22. Donnally CJ, Butler AJ, Rush AJ, Bondar KJ, Wang MY, Eismont FJ. The most influential publications in cervical myelopathy. *J Spine Surg*. 2018;4(4):770-779. doi:10.21037/jss.2018.09.08

23. Fehlings MG, Tetreault LA, Riew KD, et al. A Clinical Practice Guideline for the Management of Patients With Degenerative Cervical Myelopathy: Recommendations for Patients With Mild, Moderate, and Severe Disease and Nonmyelopathic Patients With Evidence of Cord Compression. *Global Spine J*. 2017;7(3 Suppl):70S-83S. doi:10.1177/2192568217701914

24. Deora H, Kim SH, Behari S, et al. Anterior Surgical Techniques for Cervical Spondylotic Myelopathy: WFNS Spine Committee Recommendations. *Neurospine*. 2019;16(3):408-420. doi:10.14245/ns.1938250.125

25. Zileli M. Recommendations of WFNS Spine Committee. *Neurospine*. 2019;16(3):383-385. doi:10.14245/ns.19int003

26. Zileli M, Borkar SA, Sinha S, et al. Cervical Spondylotic Myelopathy: Natural Course and the Value of Diagnostic Techniques -WFNS Spine Committee Recommendations. *Neurospine*. 2019;16(3):386-402. doi:10.14245/ns.1938240.120

For peer review only

27. Bajamal AH, Kim SH, Arifianto MR, et al. Posterior Surgical Techniques for Cervical Spondylotic Myelopathy: WFNS Spine Committee Recommendations. *Neurospine*. 2019;16(3):421-434. doi:10.14245/ns.1938274.137

28. Newson R, King L, Rychetnik L, et al. A mixed methods study of the factors that influence whether intervention research has policy and practice impacts: perceptions of Australian researchers. *BMJ Open*. 2015;5(7):e008153. doi:10.1136/bmjopen-2015-008153

29. Tetreault L, Nater A, Garwood P, Badhiwala JH, Wilson JR, Fehlings MG. Development and Implementation of Clinical Practice Guidelines: An Update and Synthesis of the Literature With a Focus in Application to Spinal Conditions. *Global Spine Journal*. 2019;9(1_suppl):53S-64S. doi:10.1177/2192568219831689

30. Hilton B, Tempest-Mitchell J, Davies BM, et al. Cord compression defined by MRI is the driving factor behind the decision to operate in Degenerative Cervical Myelopathy despite poor correlation with disease severity. *PLoS One*. 2019;14(12):e0226020. doi:10.1371/journal.pone.0226020

31. Grodzinski B, Bestwick H, Bhatti F, et al. Research activity amongst DCM research priorities. *Acta Neurochir (Wien)*. 2021;163(6):1561-1568. doi:10.1007/s00701-021-04767-6

32. Hilton B, Tempest-Mitchell J, Davies B, Kotter M. Route to diagnosis of degenerative cervical myelopathy in a UK healthcare system: a retrospective cohort study. *BMJ Open*. 2019;9(5):e027000. doi:10.1136/bmjopen-2018-027000

33. Straus SE, Tetroe J, Graham I. Defining knowledge translation. *CMAJ*. 2009;181(3-4):165-168. doi:10.1503/cmaj.081229

34. McKeen DM, Banfield JC, McIsaac DI, et al. Top ten priorities for anesthesia and perioperative research: a report from the Canadian Anesthesia Research Priority Setting Partnership. *Can J Anaesth*. 2020;67(6):641-654. doi:10.1007/s12630-020-01607-6

35. Nouri A, Tetreault L, Singh A, Karadimas SK, Fehlings MG. Degenerative Cervical Myelopathy: Epidemiology, Genetics, and Pathogenesis. *Spine (Phila Pa 1976)*. 2015;40(12):E675-693. doi:10.1097/BRS.0000000000000913

36. Nouri A, Cheng JS, Davies B, Kotter M, Schaller K, Tessitore E. Degenerative Cervical Myelopathy: A Brief Review of Past Perspectives, Present Developments, and Future Directions. *J Clin Med*. 2020;9(2):535. doi:10.3390/jcm9020535

37. Amano T, González-Varo JP, Sutherland WJ. Languages Are Still a Major Barrier to Global Science. *PLOS Biology*. 2016;14(12):e2000933. doi:10.1371/journal.pbio.2000933

38. Straus SE, Tetroe JM, Graham ID. Knowledge translation is the use of knowledge in health care decision making. *J Clin Epidemiol*. 2011;64(1):6-10. doi:10.1016/j.jclinepi.2009.08.016

39. Harrison MB, Légaré F, Graham ID, Fervers B. Adapting clinical practice guidelines to local context and assessing barriers to their use. *CMAJ*. 2010;182(2):E78-E84. doi:10.1503/cmaj.081232

For peer review only

40. Harrigan MR, Jansen JO, Schnurman Z. Knowledge silos in pediatric blunt cerebrovascular injury. *Journal of Neurosurgery: Pediatrics*. 2020;27(2):239-240. doi:10.3171/2020.8.PEDS20636

41. Schnurman Z, Golfinos JG, Roland JT, Kondziolka D. Knowledge silos: assessing knowledge sharing between specialties through the vestibular schwannoma literature. *Journal of Neurosurgery*. 2017;129(5):1278-1285. doi:10.3171/2017.6.JNS171182

42. Zwarenstein M, Reeves S. Working together but apart: barriers and routes to nurse--physician collaboration. *Jt Comm J Qual Improv*. 2002;28(5):242-247, 209. doi:10.1016/s1070-3241(02)28024-4

43. Shuldham C. A review of the impact of pre-operative education on recovery from surgery. *Int J Nurs Stud*. 1999;36(2):171-177. doi:10.1016/s0020-7489(99)00010-3

44. Wong J, Wong S. A randomized controlled trial of a new approach to preoperative teaching and patient compliance. *International Journal of Nursing Studies*. 1985;22(2):105-115. doi:10.1016/0020-7489(85)90021-5

45. Devine EC. Effects of psychoeducational care for adult surgical patients: a meta-analysis of 191 studies. *Patient Educ Couns*. 1992;19(2):129-142. doi:10.1016/0738-3991(92)90193-m

46. Safeer RS, Keenan J. Health Literacy: The Gap Between Physicians and Patients. *AFP*. 2005;72(3):463-468.

47. Khan DZ, Hazenbiller O, Gronlund T, et al. The AO Spine RECODE-DCM International Collaborative—Establishing the Foundations for Accelerated and Patient-Centered Innovation. *Global Spine J*. 2022;12(1 Suppl):159S-171S. doi:10.1177/21925682211035381

For peer review only

Figure Captions

Figure 1: Number of stakeholders that submitted questions, by region of stakeholder. Spinal surgeons were mostly based in Europe and Asia, while PwCM were much more likely to be from the UK or USA.

For peer review only

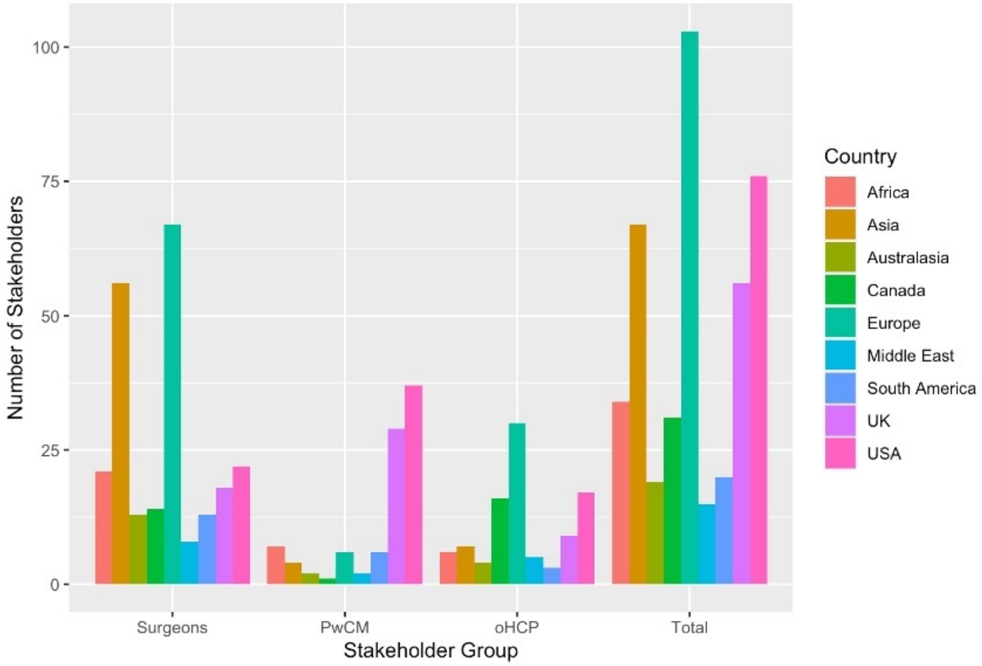

Figure 1: Number of stakeholders that submitted questions, by region of stakeholder. Spinal surgeons were mostly based in Europe and Asia, while PwCM were much more likely to be from the UK or USA.

150x102mm (144 x 144 DPI)

Supplementary Materials

Supplementary Material 1: Proportion of respondents of male sex submitting answered (Grey) and unanswered research questions (Orange), by principal stakeholder group and overall (black and red). Whilst male PwCM or their supporters were less likely to submit an answered research question, overall this association changed. It is likely this was driven by an interaction with Spinal Surgeons, who were almost universally male.

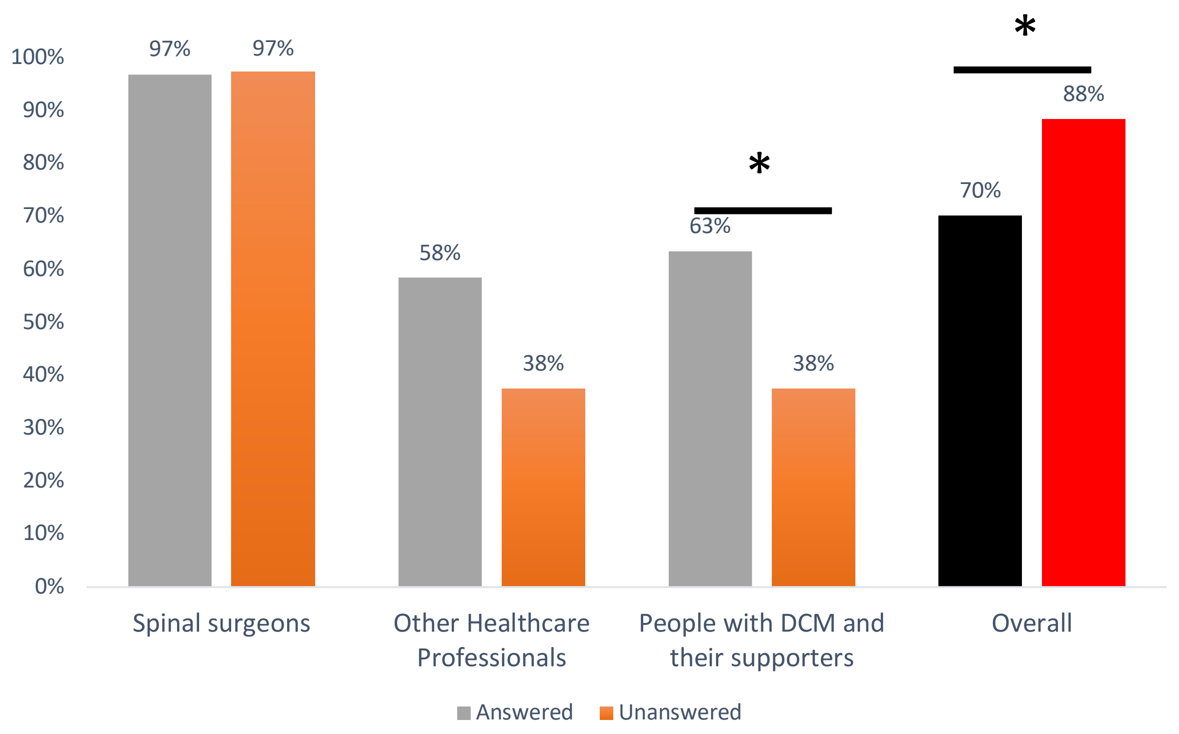

view only

For peer review only

BMJ Open

Secondary analysis of a James Lind Alliance priority setting partnership to facilitate knowledge translation in degenerative cervical myelopathy (DCM): Insights from AO Spine RECODE-DCM

Journal:	*BMJ Open*
Manuscript ID	bmjopen-2022-064296.R2
Article Type:	Original research
Date Submitted by the Author:	14-Jun-2023
Complete List of Authors:	Davies, Benjamin; University of Cambridge Neurosurgery, Department of Clinical Neurosurgery; University of Cambridge, University of Cambridge<br>Brannigan , Jamie ; University of Cambridge School of Clinical Medicine,<br>Mowforth, Oliver; University of Cambridge, Department of Academic Neurosurgery<br>Khan, Danyal; University College London,<br>McNair, Angus; University of Bristol, Centre for Surgical Research, Bristol Medical School: Population Health Sciences; North Bristol NHS Trust, GI Surgery<br>Tetreault, Lindsay; University of Toronto, Department of Surgery; University College Cork, Department of Medicine<br>Sadler, Iwan; Myelopathy.org<br>Sarewitz, Ellen; Myelopathy.org<br>Aarabi, Bizhan; University of Maryland Baltimore, Division of Neurosurgery,<br>Kwon, Brian; University of British Columbia, Division of Spine Surgery, Vancouver General Hospital<br>Gronlund, Toto ; National Institute for Health Research, University of Southampton, Southampton, UK<br>Rahimi-Movaghar, Vafa; Sina Trauma and Surgery Research Center, Academic Department of Neurological Surgery<br>Zipser, Carl Moritz; University Hospital Balgrist, Neurology<br>Hutchinson, Peter; University of Cambridge, Academic Neurosurgery<br>Kurpad, Shekar; Medical College of Wisconsin, Division of Neurosurgery<br>Harrop, James; Jefferson Health System,<br>Wilson, Jefferson R.; University of Toronto<br>Guest, James; University of Miami Miller School of Medicine, Department of Neurological Surgery<br>Fehlings, Michael G.; Toronto Western Hospital,<br>Kotter, Mark R.N.; University of Cambridge, Department of Clinical Neurosurgery; University of Cambridge
**Primary Subject Heading**:	Evidence based practice
Secondary Subject Heading:	Neurology, Surgery, Medical education and training, Qualitative research
Keywords:	NEUROSURGERY, Spine < ORTHOPAEDIC & TRAUMA SURGERY,

	SURGERY

SCHOLARONE™
Manuscripts

I, the Submitting Author has the right to grant and does grant on behalf of all authors of the Work (as defined in the below author licence), an exclusive licence and/or a non-exclusive licence for contributions from authors who are: i) UK Crown employees; ii) where BMJ has agreed a CC-BY licence shall apply, and/or iii) in accordance with the terms applicable for US Federal Government officers or employees acting as part of their official duties; on a worldwide, perpetual, irrevocable, royalty-free basis to BMJ Publishing Group Ltd ("BMJ") its licensees and where the relevant Journal is co-owned by BMJ to the co-owners of the Journal, to publish the Work in this journal and any other BMJ products and to exploit all rights, as set out in our licence.

The Submitting Author accepts and understands that any supply made under these terms is made by BMJ to the Submitting Author unless you are acting as an employee on behalf of your employer or a postgraduate student of an affiliated institution which is paying any applicable article publishing charge ("APC") for Open Access articles. Where the Submitting Author wishes to make the Work available on an Open Access basis (and intends to pay the relevant APC), the terms of reuse of such Open Access shall be governed by a Creative Commons licence – details of these licences and which Creative Commons licence will apply to this Work are set out in our licence referred to above.

Other than as permitted in any relevant BMJ Author's Self Archiving Policies, I confirm this Work has not been accepted for publication elsewhere, is not being considered for publication elsewhere and does not duplicate material already published. I confirm all authors consent to publication of this Work and authorise the granting of this licence.

For peer review only

Secondary analysis of a James Lind Alliance priority setting partnership to facilitate knowledge translation in degenerative cervical myelopathy (DCM): Insights from AO Spine RECODE-DCM

Benjamin M. Davies M.R.C.S, B.Sc. [1], Jamie F.M. Brannigan B.A.(Hons). M.B. BChir[1], Oliver D. Mowforth B.A.(Hons). M.B. BChir. [1], Danyal Z. Khan MB, BCh, BAO [1], Angus G.K. McNair, F.R.C.S Ph.D FHEA[2], Lindsay Tetreault Ph.D.[3], Iwan Sadler [4], Ellen Sarewitz M.A [5], Bizhan Aarabi MD [6], Brian K Kwon M.D. Ph.D F.R.C.S.C [7], Toto Gronlund BSc, MSc [8], Vafa Rahimi-Movaghar MD[9], Carl Moritz Zipser MD FEBN[10], Peter J.A. Hutchinson Ph.D F.R.C.S FMedSci[1], Shekar N Kurpad M.D Ph.D[11], James Harrop M.D. [12], Jefferson R Wilson M.D. Ph.D F.R.C.S.C [3], James D. Guest, MD PhD FACS [13], Michael G. Fehlings M.D. Ph.D F.R.C.S.C [3], Mark R.N. Kotter M.D. M.Phil. Ph.D. [1]

1) Academic Neurosurgery Unit, Department of Clinical Neurosurgery, University of Cambridge, Cambridge, UK
2) Center for Surgical Research, Bristol Medical School: Population Health Sciences, University of Bristol, UK
3) Division of Neurosurgery and Spine Program, Department of Surgery, University of Toronto, Toronto, Ontario, Canada
4) Myelopathy.org, Cambridge, United Kingdom. (Registered Charity England and Wales, No 1178673)
5) Person with DCM and Director of Goffin Consultancy, Healthcare Consultants.
6) Division of Neurosurgery, Shock Trauma, University of Maryland, Baltimore, Maryland, USA
7) Division of Spine Surgery, Vancouver General Hospital, University of British Columbia, Vancouver, British Columbia, Canada
8) National Institute for Health Research, University of Southampton, Southampton, UK
9) Department of Neurosurgery, Sina Trauma and Surgery Research Center, Tehran University of Medical Sciences, Tehran, Iran.
10) University Spine Center, Balgrist University Hospital, Zurich, Switzerland.

For peer review only

11) Department of Neurosurgery, Medical College of Wisconsin, Milwaukee, Wisconsin, USA
12) Division of Neurosurgery, Thomas Jefferson University Hospital, Philadelphia, Pennsylvania, USA
13) Department of Neurosurgery and The Miami Project to Cure Paralysis, The Miller School of Medicine, University of Miami, Miami, FL, United States.

Key words:

Cervical, Myelopathy, OPLL, Spondylosis, Disc Herniation, Cervical Stenosis, protocol, outcome, dataset, Core outcomes in effectiveness trials (COMET), James Lind Alliance (JLA), research priorities, Delphi, consensus, audit, surveillance, common data elements (CDE)

Running title:

Evaluation of Knowledge Translation of Surgery for Degenerative Cervical Myelopathy

Abstract word count: 171

Text word count: 3320

Number of references: 47

Number of figures/tables: 5

Correspondence to:
Dr Benjamin Davies
Division of Neurosurgery
Department of Clinical Neurosciences
University of Cambridge
bd375@cam.ac.uk
Telephone: +44 1223 763366
Fax: +44 1223 763350

For peer review only

Abstract

Objectives

To explore whether a James Lind Alliance Priority Setting Partnership could provide insights on knowledge translation within the field of degenerative cervical myelopathy (DCM).

Design

Secondary analysis of a James Lind Alliance Priority Setting Partnership process for DCM.

Participants and setting

DCM stake holders, including spinal surgeons, people with myelopathy, and other healthcare professionals were surveyed internationally. Research suggestions submitted by stakeholders but considered answered were identified. Sampling characteristics of respondents were compared to the overall cohort to identify subgroups underserved by current knowledge translation.

Results

The survey was completed by 423 individuals from 68 different countries. A total of 22% of participants submitted research suggestions that were considered 'answered'. There was a significant difference between responses from different stakeholder groups ($p<0.005$). Spinal surgeons were the group which was most likely to submit an 'answered' research question. Respondents from South America were also most likely to submit 'answered' questions, when compared to other regions. However, there was no significant difference between responses from different stakeholder regions ($p=0.4$).

Conclusions

Knowledge translation challenges exist within degenerative cervical myelopathy. This practical approach to measuring knowledge translation may offer a more responsive assessment to guide interventions, complementing existing metrics.

Strengths and limitations of this study

For peer review only

- A large number of stakeholders including patients, clinicians, and researchers were surveyed, generating 76 research questions.
- Responses came from individuals in 68 countries,
- The protocol for collecting the data analysed in this study has been published previously.
- The dissemination of the online survey through national organizations and research networks makes this study vulnerable to response bias.

For peer review only

Introduction

Degenerative Cervical Myelopathy [DCM] is the most common cause of spinal cord dysfunction worldwide, affecting up to 2% of adults[1,2]. It arises when arthritic and/or congenital changes in the cervical spine cause progressive damage and injury to the cervical spinal cord. Today, despite treatment, most patients with DCM will be left with some disability. This is often due to missed or late diagnosis[3]. Furthermore, a recent comparative study demonstrated that people with DCM have amongst the lowest quality of life scores of chronic diseases[4,5]. Consequently, urgent progress is required.

To facilitate this, AO Spine Research objectives and Common Data Elements for Degenerative Cervical Myelopathy [RECODE-DCM] (aospine.org/recode), a multi-stakeholder consensus process was undertaken. This process aimed to accelerate research progress by defining key pieces of information which can better help individual studies deliver changes in care. It combined a number of consensus initiatives, including a James Lind Alliance Priority Setting Partnership (PSP), to establish the top 10 research uncertainties[6–8].

Whilst an improvement in outcomes will require further scientific advance and clinical research, for individuals to benefit from any such progress, new knowledge must also transfer into clinical practice[9]. This transfer of knowledge, or knowledge translation [KT], is not straightforward[10], and has been reported to take well over a decade in some cases[11]. For people with DCM, effective knowledge translation could be considered as important as knowledge discovery. This would be reflected in their selection of ‘raising awareness’ as the number one research priority for DCM[12].

A variety of strategies and frameworks have been proposed to optimize the KT process[13,14], including the formation of clinical practice guidelines. However, commonly this process

For peer review only

requires active surveillance and iteration. To that end, approaches to measure knowledge uptake are important but less well defined.

The aim of a PSP (Priority Setting Partnership) is to identify the critical knowledge gaps. This starts by seeking research suggestions from both people who have and who treat a condition (e.g., DCM), across relevant healthcare disciplines. These submissions are then processed and consolidated into summary questions. Each summary question is evaluated against the current evidence base and is removed from the process if it is felt to have already been answered. The remaining questions are then taken forward to be prioritized[8]. These steps for AO Spine RECODE DCM has been previously described[8].

Here, we explored the concept that the questions submitted by individuals as being “ongoing research questions” but considered otherwise to have been answered, might highlight areas where knowledge translation was particularly lacking.

For peer review only

Method

Survey

The protocol for AO Spine RECODE-DCM is published in the Global Spine Journal[15]. DCM stakeholders were recruited to an internet survey hosted by Calibrum (California, USA). AO Spine RECODE-DCM identified three principal stakeholder groups to partake in this initiative: Spinal Surgeons, Persons with DCM [PwCM] and their family or friends, and other healthcare professionals [oHCPs], including neurologists and physiotherapists.

A detailed summary of the dissemination process has been published[7]. An international contact directory was compiled of DCM stakeholder individuals and organisations. The directory comprised a list of names and contact email addresses for stakeholder individuals such as neurosurgeons, orthopaedic surgeons, neurologists, general practitioners and physiotherapists. Contact details for stakeholder organisations were also collected, including medical charities, universities, medical colleges, hospitals and medical journals. An email campaign targeted at stakeholders in the contact directory was executed using MailChimp (Georgia, US). Emails provided a concise introduction to AO Spine RECODE-DCM, explained that we had identified the individual as someone who may be interested in participating, and provided a link to the survey. A total of 5 emails were sent to the global contact directory, each separated by approximately 1 week.

Respondents were randomised to a core outcome set stream and a PSP stream. In the PSP stream, participants were invited to enter as free text what they thought were the most important DCM research questions within each of the 4 categories of diagnosis, treatment, long-term care and follow-up and other.

The survey was closed at the point of information saturation, defined as no additional unique research suggestions at a two-week interval. Following closure of the survey, research

For peer review only

suggestions were processed by an information specialist[8,15]. Suggestions were grouped into common themes which were then used to form representative summary questions. All summary questions underwent an evidence checking process, including search of the literature and discussion with the Steering Committee. Questions were defined as either ‘unanswered’ or ‘answered’ depending on whether there was sufficient quality of evidence available in the literature. Scoping reviews of the literature were conducted by LT, the designated information specialist for this JLA PSP, to find evidence to support this process. Questions that were considered ‘answered’ were removed from the process following review and discussion with the Steering Committee, composed of 6 neurosurgeons, 1 orthopedic surgeon, 2 neurologists, 1 primary care physician, 3 rehabilitation specialists and 12 PwCM[8].

Of the 76 summary questions generated, two were considered to have been answered: (1) *What is the safety and efficacy of surgical interventions for DCM?* and (2) *What is the efficacy and safety of anterior versus posterior surgery in patients with DCM?* The decision to remove this latter question also considered that Cervical Spondylotic Myelopathy Surgical Trial (CSM-S, NCT02076113), a randomized controlled trial of anterior vs. posterior surgery, was in process. For brevity these will be referred to as the ‘Effectiveness’ and ‘Anterior vs. Posterior’ questions. The remaining 74 questions, which were considered unanswered, are publicly available on the James Lind Alliance PSP website[16].

Analysis

Demographics of participants who submitted ‘answered’ and ‘unanswered’ summary questions were aggregated for analysis. For healthcare professionals, this included specialty, experience with DCM, age and country of employment. For PwCM or their supporters, this included country of residence and years lived with DCM. All participants were asked to provide their age and biological sex.

For peer review only

Geography is often an important consideration for knowledge translation for many reasons, including language, applicability to local practice and the physical barrier it can create for information exchange[17]. To explore this, country of residence or practice were aggregated into common zones – either by country if there was sufficient representation or continent if not. Countries were further categorized as Higher Income Countries or not, using the World Bank (worldbank.org) classification [22nd October, 2020]. In addition, we and others have identified that DCM research is largely derived from two geographical clusters: North America (Canada and the USA) and East Asia (Japan, Korea and China)[17,18]. To explore a relationship between research activity and knowledge translation, participants were also defined by whether they reside or practice within a research cluster or not.

Comparisons between groups, based on factors such as region and level of experience, were made using Chi-Squared test for categorical or ordinal data, and Mann Whitney U test for continuous data. Significance was defined as $p<0.05$.

Analysis and data visualisation were performed using R (v4.0.5; R Core Team, 2020) and RStudio (v1.4.1106; RStudio Team, 2021).

Patient and public involvement

Patient and carer representatives were engaged throughout the process. They helped define the scope and were involved in the review of all patient-facing media. They were involved in all steering group meetings and decisions. They collaborated with patient organisations and helped to reach a diverse range of patient and carers groups for the surveys and final workshop. Patient representatives will help disseminate the PSP findings and work with patient and charitable organisations to develop discrete research questions from the final priorities to take forward for funding.

For peer review only

Results

Summary

The survey was completed by 423 individuals from 68 different countries[7]. This included 232 surgeons (55%), 94 PwCM (22%) and 95 oHCP (23%). PwCM were principally from USA (41%) or the UK (32%). Surgeons and oHCP were more evenly distributed (Figure 1). In total, 95 (22%) participants submitted a research suggestion that mapped to one or both of these answered research questions; 51 (12%) 'Effective' and 44 (10%) 'Anterior vs Posterior'. This included 75 (32%) spinal surgeons, 12 (13%) PwCM and 8 (8%) oHCPs.

Submission of Research Suggestions that were "Answered" vs "Unanswered"

In the group that submitted a research suggestion that was deemed to be "answered" (i.e., around surgical "effectiveness" and "anterior vs posterior" surgery), there were 75 (79%) Surgeons, 12 (13%) oHCP and 8 (8%) PwCM. Spinal surgeons ($p<0.005$) and those of male sex ($p<0.005$) were more likely to submit a research suggestion that was already answered (Table 1; Supplementary Material 1).

*Table 1: Spinal Surgeon Stakeholders (N=232), Subgroup analysis. A high activity DCM research cluster was defined from a prior co-author network analysis – specifically DCM research activity clusters geographically to North America and East Asia (Japan, China and South Korea). *Significance, p<0.05.*

		Unanswered (%)		Answered (%)		P
Age		44.4		44.5		0.6
Male Gender		152	97%	73	97%	1
Region						0.21
	USA	13	8%	9	12%	
	UK	11	7%	7	9%	
	Canada	12	8%	2	3%	
	Europe	49	31%	18	24%	
	South America	6	4%	7	9%	

For peer review only

Middle East	3	2%	5	7%	
Asia	40	25%	16	21%	
Australasia	8	5%	5	7%	
Africa	15	10%	6	8%	
Research Cluster					
DCM Case Treated Yearly					0.78
0-25	28	18%	15	20%	
25-50	55	35%	24	32%	
50-100	47	30%	19	25%	
100+	27	17%	16	21%	
Year's Experience	13.6	9.7%	14.2	13.5%	
Neurosurgeon by Training	95	61%	45	60%	0.35
From a high-activity, DCM Research Cluster	43	27%	16	21%	0.41
High Income Country	111	71%	47	63%	0.28

Individuals were less likely to submit an answered research question if they resided or practiced within an active DCM research cluster (Japan, China, South Korea, USA or Canada) or high-income countries (Tables 1, 2, 3 and 4).

*Table 2: Comparison of respondent demographics of participants who submitted research suggestions that mapped to answered (N=95) compared to unanswered (N=328) summary questions. A high activity DCM research cluster was defined from a prior co-author network analysis – specifically DCM research activity clusters geographically to North America and East Asia (Japan, China and South Korea). *Significance, p<0.05.*

	Unanswered (%)		Answered (%)		P
N	328	78%	95	22%	
Stakeholder Group					*<0.005**
Spinal surgeons	157	48%	75	79%	

For peer review only

People with DCM and their supporters	82	25%	12	13%	
Other healthcare professionals	89	27%	8	8%	
Age (SD)	47.9	11.7	46.4	11.68	0.25
Male Gender	230	70%	84	88%	*<0.005*
Region					0.4
USA	62	19%	14	15%	
UK	43	13%	13	14%	
Canada	27	8%	4	4%	
Europe	82	25%	21	22%	
South America	14	4%	8	8%	
Middle East	10	3%	5	5%	
Asia	49	15%	18	19%	
Australasia	13	4%	6	6%	
Africa	28	9%	6	6%	
From a high-activity, DCM Research Cluster	109	33%	23	24%	0.12
High Income Country	250	76%	63	66%	0.07

*Table 1: Other Healthcare Professional Stakeholders (N=95), Subgroup Analysis. A high activity DCM research cluster was defined from a prior co-author network analysis – specifically DCM research activity clusters geographically to North America and East Asia (Japan, China and South Korea). *Significance, p<0.05.*

	**Unanswered (%)**		**Answered (%)**		**P**
Age	45.7		42.8		
Male Gender	52	58%	3	38%	0.4
Region					
USA	17	11%	0	0%	0.49

For peer review only

UK	7	4%	2	3%	
Canada	14	9%	2	3%	
Europe	28	18%	2	3%	
South America	3	2%	0	0%	
Middle East	5	3%	0	0%	
Asia	6	4%	1	1%	
Australasia	3	2%	1	1%	
Africa	6	4%	0	0%	
Research Cluster (Japan / China / N.America)					
DCM Volume					0.23
0-25	49	31%	7	9%	
25-50	20	13%	0	0%	
50-100	12	8%	0	0%	
100+	7	4%	1	1%	
Years Experience	14.7	9.7	9.9	13.5	0.88
From a high-activity, DCM Research Cluster	32	36%	2	25%	0.8
High Income Country	72	81%	6	75%	1
Discipline					
Neurologist	18	11%	0	0%	0.23
Physiotherapist	10	6%	1	1%	
Rehabilitation Medicine	9	6%	3	4%	
General Practitioner	9	6%	0	0%	
General Physician	10	6%	1	1%	
Other	32	20%	3	4%	

*Table 2: Persons with DCM or their supporters (friends or family), Subgroup Analysis. A high activity DCM research cluster was defined from a prior co-author network analysis – specifically DCM research activity clusters geographically to North America and East Asia (Japan, China and South Korea). *Significance, p<0.05.*

	Unanswered (%)		Answered (%)		P
Age	45.7		42.75		0.47
Male Gender	52	63%	3	38%	0.03
Region					
USA	32	39%	5	43%	0.96
UK	25	30%	4	33%	
Canada	1	1%	0	1%	
Europe	5	6%	1	7%	
South America	5	6%	1	7%	
Middle East	2	2%	0	3%	
Asia	3	4%	1	4%	
Australasia	2	2%	0	3%	
Africa	7	9%	0	9%	
Years Lived with DCM	5.5	4.8	5.3	4.2	0.8
From a high-activity, DCM Research Cluster	34	41%	10	45%	1
High Income Country	67	82%	10	89%	1

Professional experience or discipline was not associated with the likelihood of submitting an answered research question. Of note, no neurologist (N=18) submitted a research suggestion that mapped to an “answered” research question (Table 3).

Demographics were compared of those who submitted answered research suggestions, by whether it mapped to the ‘Effectiveness’ or to the ‘Anterior vs. Posterior’ questions. Spinal

For peer review only

surgeons and respondents from Asia ($p<0.05$) were more likely to submit research questions related to ‘Anterior vs. Posterior’ approaches.

For peer review only

Discussion

Knowledge translation is a major issue in DCM. This is reflected by its selection as the number one research priority by AO Spine RECODE DCM – Raising Awareness[12]. This was also reflected within this analysis, as 22% of participants submitted research suggestions that were considered 'answered'. Spinal surgeons were more likely to submit an answered research question than oHCPs or PwCM. Anterior versus posterior surgery was more likely to be suggested by surgeons and respondents from Asia. Individuals living or practicing within a higher income country, or a country with high DCM research activity, tended to be less likely to submit an answered research suggestion; this association, however, was non-significant.

Can evidence checking of research suggestions act as a KT metric and inform KT strategy?

Ultimately, this was an exploratory analysis of an existing dataset, and cannot establish whether analysis of research suggestions is truly an effective KT metric. For example, many respondents in possession of the evidence may have considered the 'Anterior vs. Posterior' question to be unanswered. Our findings may instead reflect conflicting interpretations of the evidence, rather than poor KT. In this regard it was perhaps noteworthy that this question was more likely to be submitted by Asian surgeons where OPLL is more prevalent [19]. However, the results, taken in wider context, suggest promise.

Building on the significant growth in DCM research[20] and clinical evidence[21,22], clinical practice guidelines for DCM have been developed by AO Spine[23] and the World Federation of Neurosurgeons (WFNS) separately[24–27]. Whilst there remain many unanswered questions in DCM[15], these guidelines consolidate the current evidence on the effectiveness of surgical treatment[23]. Guidelines are considered one of the most effective tools for knowledge

For peer review only

translation [28,29]. Despite this, an audit of surgical practice has shown poor adherence to these guidelines[30], and DCM research continues to be dominated by investigations into these 'answered' research questions by surgeons[31]. This would suggest an ongoing KT gap and would align with the observed 75 (33%) surgeons who submitted at least one 'answered' question relating to this. This would also align with the on average 10-15 years[11] taken to bring new knowledge into routine practice.

Efforts to support the dissemination of these guidelines and inform evidence-based care are on-going. One of the challenges is the large number of specialities currently coordinating DCM care – all potential target audiences, e.g. general practice, neurology, physiotherapy, orthopedics, neurosurgery, rheumatology, gerontologists, and rehabilitation physicians[32]. Although the scope of answered questions was restricted to surgery, these research suggestions were still submitted by 8% of oHCP and 13% of PwCM; 8% and 13%.

The success of KT, or strategies to accelerate KT, are conventionally assessed through changes to guidelines, surveys of care providers and measurement of service/product/pathway adoption, where applicable[33]. Whilst valid, each of these metrics take considerable time to perform and, often, for example with respect to uptake within guidelines, would lag considerably a KT intervention. This means the recognition of successful or failed strategies, and/or need to iterate KT strategies, often is not very responsive.

Our experience here would suggest the assessment of the 'answered' research suggestions could offer a live snapshot of KT progress, concerning both patients and clinicians. Clearly its application may not be appropriate in all settings. For example, this analysis approach could not be applied to a PSP for perioperative care in Canada, where no research suggestions were deemed to be answered already[34]. This approach will also be vulnerable to selection bias. For example, as was the case in AO Spine RECODE DCM, engaging

For peer review only

stakeholders through electronic surveys outside of spinal surgery was very difficult. However, efforts could be taken to mitigate this.

Further, the relative differences in sampling characteristics may be helpful. In this study, participants were less likely to submit an answered research question if they came from a high-income country, or a country with higher DCM research activity. Questions relating to Anterior vs Posterior surgery were also more likely to come from Asia. Overall, these differences may indicate groups underserved by current KT strategies.

Factors contributing to Knowledge Translation gaps in DCM

There are several proposed barriers to rapid dissemination of DCM knowledge:

Terminology – First proposed in 2015[35], the umbrella term of DCM is still not universal[36]. 'Cervical spondylotic myelopathy' is the most commonly used term, but this has an inconsistent definition[36]. The use of variable terminology may therefore impede KT.

Geography/language – the major international guidelines have been published in English[23]. While this may not affect our study this affects international adoption of new knowledge[37].

Adaptation for local use - adaptation of knowledge to a local context is a key step in the knowledge-to-action cycle[38]. This is a pro-active process which must take place in individual hospitals and hospital networks[39].

There are also further barriers to the transfer of knowledge between different stakeholder groups.

For oHCPs, 'knowledge silos' have been described over the last decade between specialties[40,41]. The existence of different journals, vocabulary, professional organizations, and priorities are all believed to contribute to this[40,41]. Silos act to from closed-communication

For peer review only

loops which inhibit knowledge diffusion. We expect that this applies to surgeons, neurologists, and general practitioners in the case of DCM. Impaired collaboration also exists between clinicians and allied health professionals[42], which may form a barrier to KT within specialties.

KT to PwCM is also significant. If considering knowledge about surgical interventions, it has been long established that improved patient knowledge in the pre-operative phase enhances post-operative outcomes[43]. This is true in several domains, including post-operative compliance[44] and subjective pain reporting[45]. Barriers to the transfer of knowledge to PwCM include clinician knowledge and health literacy[46].

The relative comparison between 'answered' questions submitted by different stakeholder groups may reveal further insights about KT and the strategies used to tackle the above barriers. It is hoped the emergence of the RECODE-DCM community may also become a tool to address this[47].

Conclusion and Future Directions

Answered research questions were frequently submitted during the AO Spine RECODE DCM Priority Setting Partnership, indicating a knowledge translation problem in DCM. This practical approach to measuring knowledge translation may more widely offer a responsive assessment to guide interventions, complementing existing KT metrics which provide retrospective assessments. In the future, knowledge translation in AOSpine RECODE-DCM will need to involve considerable outreach to the broader community of health care providers involved with DCM, health care funders and policy makers and the public.

For peer review only

Ethics Statement

Ethical approval for the study was granted by the Human Biology Research Ethics Committee, University of Cambridge (HBREC.2019.14). All patients involved provided informed consent.

Contributors

BD, OM, DK, AM, LT, IS, ES, BA, BK, TG, VRM, CZ, PH, SK, JH, JW, JG, MF and MK were involved in the conceptualisation and design of this study. BD and JB wrote the first draft of the manuscript. BD, JB, OM, LT, BK and MF were involved in revising the manuscript. BD, JB, OM, DK, AM, LT, IS, ES, BA, BK, TG, VRM, CZ, PH, SK, JH, JW, JG, MF and MK reviewed and approved the final manuscript.

Competing interests

BD, JB, OM, IS, ES, MF and MK have voluntary roles at Myelopathy.org, an international DCM charity.

Funding

NIHR Clinician Scientist Fellowship, Award/Grant no: NIHR-CS-2017-17-010 (AM)

NIHR Clinician Scientist Award, Award/Grant no: CS-2015-15-023 (MRNK)

NIHR Research Fellowship, Award/Grant no: NA (BMD)

Disclaimer

The views expressed in this publication are those of the authors and not necessarily those of the NHS, the National Institute for Health Research or the Department of Health.

Data availability statement

For peer review only

Data are available upon reasonable request from the corresponding author.

For peer review only

Acknowledgements

The research priorities were organized and funded by AO Spine through the AO Spine Knowledge Forum Spinal Cord Injury, a focused group of international Spinal Cord Injury experts. AO Spine is a clinical division of the AO Foundation, which is an independent medically-guided not-for-profit organization. Study support was provided directly through the AO Spine Research Department.

This research aligns with the AO Spine RECODE-DCM James Lind Alliance top research priority Raising Awareness selected by people living and working with DCM. For further information on how this process was conducted, why this question was prioritised, and global updates on currently aligned research, please visit aospine.org/recode/raising-awareness.

We thank Dr Joanna Crocker (Interdisciplinary Research in Health Sciences, University of Oxford) for her help with our methodology and statistical reporting.

AM is funded by a Clinician Scientist Fellowship (NIHR-CS-2017-17-010) from the UK National Institute for Health Research (NIHR) and supported by the NIHR Biomedical Research Centre at the University Hospitals Bristol NHS Foundation Trust and the University of Bristol. MRNK is supported by a NIHR Clinician Scientist Award, CS-2015-15-023. BMD is supported by a research fellowship from the Royal College of Surgeons (London), and the NIHR, United Kingdom. The views expressed in this publication are those of the authors and not necessarily those of the NHS, the National Institute for Health Research or the Department of Health.

For peer review only

References

1. Davies BM, Mowforth OD, Smith EK, Kotter MR. Degenerative cervical myelopathy. *BMJ*. 2018;360:k186. doi:10.1136/bmj.k186

2. Smith SS, Stewart ME, Davies BM, Kotter MRN. The Prevalence of Asymptomatic and Symptomatic Spinal Cord Compression on Magnetic Resonance Imaging: A Systematic Review and Meta-analysis. *Global Spine Journal*. 2021;11(4):597-607. doi:10.1177/2192568220934496

3. Grodzinski B, Stubbs DJ, Davies BM. Most degenerative cervical myelopathy remains undiagnosed, particularly amongst the elderly: modelling the prevalence of degenerative cervical myelopathy in the United Kingdom. *J Neurol*. Published online September 2, 2022. doi:10.1007/s00415-022-11349-8

4. Pope DH, Mowforth OD, Davies BM, Kotter MRN. Diagnostic Delays Lead to Greater Disability in Degenerative Cervical Myelopathy and Represent a Health Inequality. *Spine (Phila Pa 1976)*. 2020;45(6):368-377. doi:10.1097/BRS.0000000000003305

5. Oh T, Lafage R, Lafage V, et al. Comparing Quality of Life in Cervical Spondylotic Myelopathy with Other Chronic Debilitating Diseases Using the Short Form Survey 36-Health Survey. *World Neurosurg*. 2017;106:699-706. doi:10.1016/j.wneu.2016.12.124

6. Cowan K. The James Lind alliance: tackling treatment uncertainties together. *J Ambul Care Manage*. 2010;33(3):241-248. doi:10.1097/JAC.0b013e3181e62cda

7. Mowforth OD, Khan DZ, Wong MY, et al. Gathering Global Perspectives to Establish the Research Priorities and Minimum Data Sets for Degenerative Cervical Myelopathy: Sampling Strategy of the First Round Consensus Surveys of AO Spine RECODE-DCM. *Global Spine J*. Published online December 8, 2021:21925682211047544. doi:10.1177/21925682211047546

8. Tetreault L, Mowforth O, Khan DZ, et al. James Lind Alliance Priority Setting Partnership for Degenerative Cervical Myelopathy [AO Spine RECODE-DCM]: An Overview of the Methodology Used to Process and Short-List Research Uncertainties. *Global Spine Journal*. 2022;12(1_suppl):19S-27S. doi:10.1177/21925682211062501

9. Djulbegovic B, Guyatt GH. Progress in evidence-based medicine: a quarter century on. *Lancet*. 2017;390(10092):415-423. doi:10.1016/S0140-6736(16)31592-6

10. Linskey ME. Evidence-Based Medicine for Neurosurgeons: Introduction and Methodology. *Guiding Neurosurgery by Evidence*. 2006;19:1-53. doi:10.1159/000095175

11. Morris ZS, Wooding S, Grant J. The answer is 17 years, what is the question: understanding time lags in translational research. *J R Soc Med*. 2011;104(12):510-520. doi:10.1258/jrsm.2011.110180

12. Davies BM. Improving awareness could transform outcomes in Degenerative Cervical Myelopathy (DCM) [AO Spine RECODE DCM Research Priority Number 1]. *Global Spine Journal*. Published online 2022.

13. Graham ID, Logan J, Harrison MB, et al. Lost in knowledge translation: time for a map? *J Contin Educ Health Prof*. 2006;26(1):13-24. doi:10.1002/chp.47

For peer review only

14. Grol R, Grimshaw J. From best evidence to best practice: effective implementation of change in patients' care. *The Lancet*. 2003;362(9391):1225-1230. doi:10.1016/S0140-6736(03)14546-1

15. Davies BM, Khan DZ, Mowforth OD, et al. RE-CODE DCM (REsearch Objectives and Common Data Elements for Degenerative Cervical Myelopathy): A Consensus Process to Improve Research Efficiency in DCM, Through Establishment of a Standardized Dataset for Clinical Research and the Definition of the Research Priorities. *Global Spine Journal*. 2019;9(1_suppl):65S-76S. doi:10.1177/2192568219832855

16. Degenerative Cervical Myelopathy | James Lind Alliance. Accessed December 15, 2022. https://www.jla.nihr.ac.uk/priority-setting-partnerships/Degenerative-Cervical-Myelopathy/

17. Duan Y, Nie W, Coakes E. Identifying key factors affecting transnational knowledge transfer. Published online December 2010. doi:10.1016/j.im.2010.08.003

18. Grodzinski N, Grodzinski B, Davies BM. Can co-authorship networks be used to predict author research impact? A machine-learning based analysis within the field of degenerative cervical myelopathy research. *PLoS One*. 2021;16(9):e0256997. doi:10.1371/journal.pone.0256997

19. Choi BW, Song KJ, Chang H. Ossification of the Posterior Longitudinal Ligament: A Review of Literature. *Asian Spine J*. 2011;5(4):267-276. doi:10.4184/asj.2011.5.4.267

20. Mowforth OD, Davies BM, Goh S, O'Neill CP, Kotter MRN. Research Inefficiency in Degenerative Cervical Myelopathy: Findings of a Systematic Review on Research Activity Over the Past 20 Years. *Global Spine Journal*. 2020;10(4):476-485. doi:10.1177/2192568219847439

21. Gnanalingham KK, Davies BM, Balamurali G, Titoria P, Doyle P, Abou-Zeid A. Improving levels of evidence in studies published in spinal journals from 1983 to 2011. *British Journal of Neurosurgery*. 2013;27(2):152-155. doi:10.3109/02688697.2012.737957

22. Donnally CJ, Butler AJ, Rush AJ, Bondar KJ, Wang MY, Eismont FJ. The most influential publications in cervical myelopathy. *J Spine Surg*. 2018;4(4):770-779. doi:10.21037/jss.2018.09.08

23. Fehlings MG, Tetreault LA, Riew KD, et al. A Clinical Practice Guideline for the Management of Patients With Degenerative Cervical Myelopathy: Recommendations for Patients With Mild, Moderate, and Severe Disease and Nonmyelopathic Patients With Evidence of Cord Compression. *Global Spine J*. 2017;7(3 Suppl):70S-83S. doi:10.1177/2192568217701914

24. Deora H, Kim SH, Behari S, et al. Anterior Surgical Techniques for Cervical Spondylotic Myelopathy: WFNS Spine Committee Recommendations. *Neurospine*. 2019;16(3):408-420. doi:10.14245/ns.1938250.125

25. Zileli M. Recommendations of WFNS Spine Committee. *Neurospine*. 2019;16(3):383-385. doi:10.14245/ns.19int003

26. Zileli M, Borkar SA, Sinha S, et al. Cervical Spondylotic Myelopathy: Natural Course and the Value of Diagnostic Techniques -WFNS Spine Committee Recommendations. *Neurospine*. 2019;16(3):386-402. doi:10.14245/ns.1938240.120

For peer review only

27. Bajamal AH, Kim SH, Arifianto MR, et al. Posterior Surgical Techniques for Cervical Spondylotic Myelopathy: WFNS Spine Committee Recommendations. *Neurospine*. 2019;16(3):421-434. doi:10.14245/ns.1938274.137

28. Newson R, King L, Rychetnik L, et al. A mixed methods study of the factors that influence whether intervention research has policy and practice impacts: perceptions of Australian researchers. *BMJ Open*. 2015;5(7):e008153. doi:10.1136/bmjopen-2015-008153

29. Tetreault L, Nater A, Garwood P, Badhiwala JH, Wilson JR, Fehlings MG. Development and Implementation of Clinical Practice Guidelines: An Update and Synthesis of the Literature With a Focus in Application to Spinal Conditions. *Global Spine Journal*. 2019;9(1_suppl):53S-64S. doi:10.1177/2192568219831689

30. Hilton B, Tempest-Mitchell J, Davies BM, et al. Cord compression defined by MRI is the driving factor behind the decision to operate in Degenerative Cervical Myelopathy despite poor correlation with disease severity. *PLoS One*. 2019;14(12):e0226020. doi:10.1371/journal.pone.0226020

31. Grodzinski B, Bestwick H, Bhatti F, et al. Research activity amongst DCM research priorities. *Acta Neurochir (Wien)*. 2021;163(6):1561-1568. doi:10.1007/s00701-021-04767-6

32. Hilton B, Tempest-Mitchell J, Davies B, Kotter M. Route to diagnosis of degenerative cervical myelopathy in a UK healthcare system: a retrospective cohort study. *BMJ Open*. 2019;9(5):e027000. doi:10.1136/bmjopen-2018-027000

33. Straus SE, Tetroe J, Graham I. Defining knowledge translation. *CMAJ*. 2009;181(3-4):165-168. doi:10.1503/cmaj.081229

34. McKeen DM, Banfield JC, McIsaac DI, et al. Top ten priorities for anesthesia and perioperative research: a report from the Canadian Anesthesia Research Priority Setting Partnership. *Can J Anaesth*. 2020;67(6):641-654. doi:10.1007/s12630-020-01607-6

35. Nouri A, Tetreault L, Singh A, Karadimas SK, Fehlings MG. Degenerative Cervical Myelopathy: Epidemiology, Genetics, and Pathogenesis. *Spine (Phila Pa 1976)*. 2015;40(12):E675-693. doi:10.1097/BRS.0000000000000913

36. Nouri A, Cheng JS, Davies B, Kotter M, Schaller K, Tessitore E. Degenerative Cervical Myelopathy: A Brief Review of Past Perspectives, Present Developments, and Future Directions. *J Clin Med*. 2020;9(2):535. doi:10.3390/jcm9020535

37. Amano T, González-Varo JP, Sutherland WJ. Languages Are Still a Major Barrier to Global Science. *PLOS Biology*. 2016;14(12):e2000933. doi:10.1371/journal.pbio.2000933

38. Straus SE, Tetroe JM, Graham ID. Knowledge translation is the use of knowledge in health care decision making. *J Clin Epidemiol*. 2011;64(1):6-10. doi:10.1016/j.jclinepi.2009.08.016

39. Harrison MB, Légaré F, Graham ID, Fervers B. Adapting clinical practice guidelines to local context and assessing barriers to their use. *CMAJ*. 2010;182(2):E78-E84. doi:10.1503/cmaj.081232

For peer review only

40. Harrigan MR, Jansen JO, Schnurman Z. Knowledge silos in pediatric blunt cerebrovascular injury. *Journal of Neurosurgery: Pediatrics*. 2020;27(2):239-240. doi:10.3171/2020.8.PEDS20636

41. Schnurman Z, Golfinos JG, Roland JT, Kondziolka D. Knowledge silos: assessing knowledge sharing between specialties through the vestibular schwannoma literature. *Journal of Neurosurgery*. 2017;129(5):1278-1285. doi:10.3171/2017.6.JNS171182

42. Zwarenstein M, Reeves S. Working together but apart: barriers and routes to nurse--physician collaboration. *Jt Comm J Qual Improv*. 2002;28(5):242-247, 209. doi:10.1016/s1070-3241(02)28024-4

43. Shuldham C. A review of the impact of pre-operative education on recovery from surgery. *Int J Nurs Stud*. 1999;36(2):171-177. doi:10.1016/s0020-7489(99)00010-3

44. Wong J, Wong S. A randomized controlled trial of a new approach to preoperative teaching and patient compliance. *International Journal of Nursing Studies*. 1985;22(2):105-115. doi:10.1016/0020-7489(85)90021-5

45. Devine EC. Effects of psychoeducational care for adult surgical patients: a meta-analysis of 191 studies. *Patient Educ Couns*. 1992;19(2):129-142. doi:10.1016/0738-3991(92)90193-m

46. Safeer RS, Keenan J. Health Literacy: The Gap Between Physicians and Patients. *AFP*. 2005;72(3):463-468.

47. Khan DZ, Hazenbiller O, Gronlund T, et al. The AO Spine RECODE-DCM International Collaborative—Establishing the Foundations for Accelerated and Patient-Centered Innovation. *Global Spine J*. 2022;12(1 Suppl):159S-171S. doi:10.1177/21925682211035381

For peer review only

Figure Captions

Figure 1: Number of stakeholders that submitted questions, by region of stakeholder. Spinal surgeons were mostly based in Europe and Asia, while PwCM were much more likely to be from the UK or USA.

For peer review only

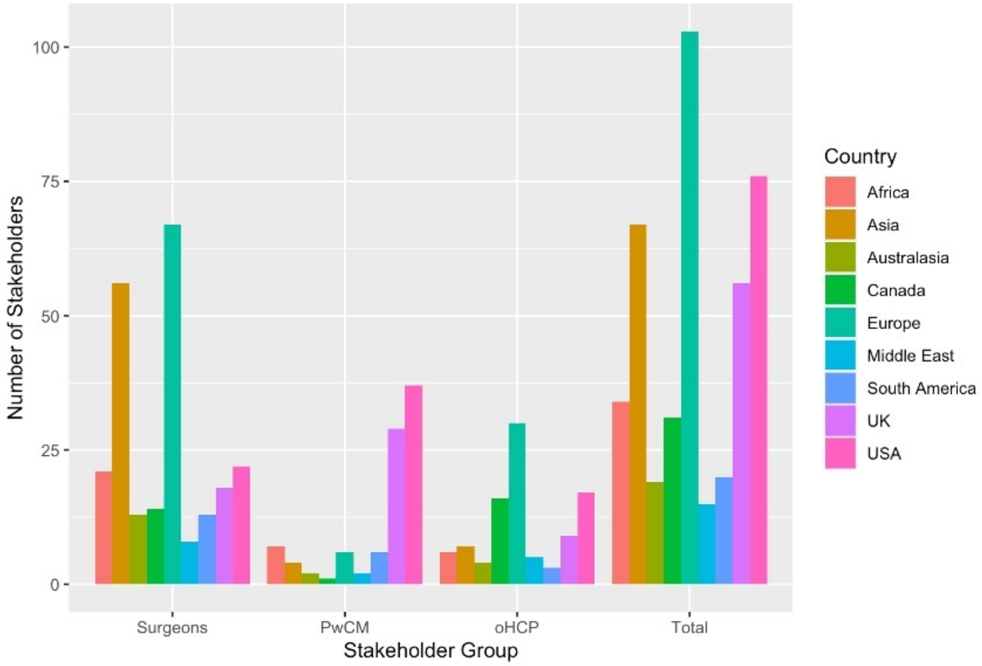

Figure 1: Number of stakeholders that submitted questions, by region of stakeholder. Spinal surgeons were mostly based in Europe and Asia, while PwCM were much more likely to be from the UK or USA.

150x102mm (144 x 144 DPI)

Supplementary Materials

Supplementary Material 1: Proportion of respondents of male sex submitting answered (Grey) and unanswered research questions (Orange), by principal stakeholder group and overall (black and red). Whilst male PwCM or their supporters were less likely to submit an answered research question, overall this association changed. It is likely this was driven by an interaction with Spinal Surgeons, who were almost universally male.

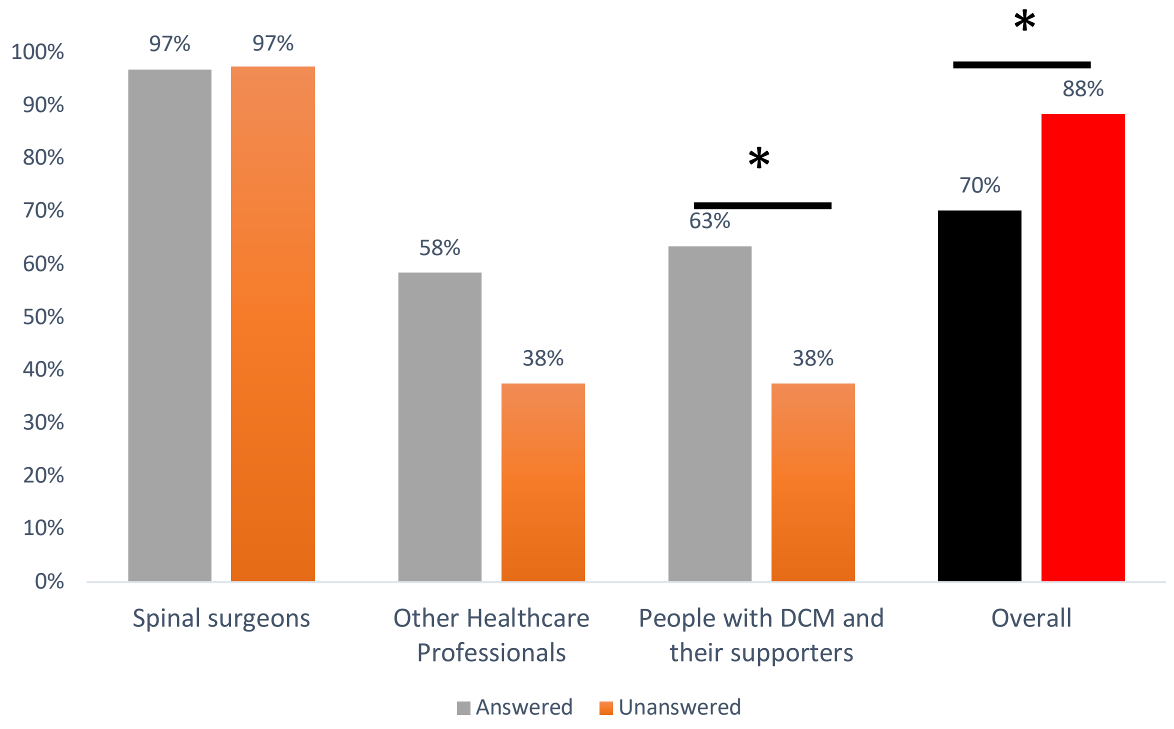

view only

For peer review only
